# Pro-inflammatory cytokines activate hypoxia-inducible factor 3α via epigenetic changes in mesenchymal stromal/stem cells

**DOI:** 10.1038/s41598-018-24221-5

**Published:** 2018-04-11

**Authors:** Francesca Cuomo, Antonietta Coppola, Chiara Botti, Ciro Maione, Amalia Forte, Lucia Scisciola, Giuseppina Liguori, Ilaria Caiafa, Matilde Valeria Ursini, Umberto Galderisi, Marilena Cipollaro, Lucia Altucci, Gilda Cobellis

**Affiliations:** 1 Department of Biochemistry, Biophysics and General Pathology, Università degli Studi della Campania L. Vanvitelli, Via L. De Crecchio, 7, 80138 Naples, Italy; 2 Department of Experimental Medicine, Università degli Studi della Campania L. Vanvitelli, Via L. De Crecchio, 7, 80138 Naples, Italy; 3 Istituto Nazionale Tumori, Struttura Complessa Oncologia Medica Melanoma Immunoterapia Oncologica e Terapia Innovativa, Via M. Semmola, 80131 Naples, Italy; 4 Institute of Genetics and Biophysics, ‘A. Buzzati-Traverso’ (IGB), via P. Castellino, 111, 80131 Naples, Italy; 5 Present Address: Laboratorio di Patologia Clinica, Ospedale Santobono, Via M. Fiore 6, 80129 Naples, Italy

**Keywords:** Epigenetics, Cardiovascular diseases

## Abstract

Human mesenchymal stromal/stem cells (hMSCs) emerged as a promising therapeutic tool for ischemic disorders, due to their ability to regenerate damaged tissues, promote angiogenesis and reduce inflammation, leading to encouraging, but still limited results. The outcomes in clinical trials exploring hMSC therapy are influenced by low cell retention and survival in affected tissues, partially influenced by lesion’s microenvironment, where low oxygen conditions (i.e. hypoxia) and inflammation coexist. Hypoxia and inflammation are pathophysiological stresses, sharing common activators, such as hypoxia-inducible factors (HIFs) and NF-κB. HIF1α and HIF2α respond essentially to hypoxia, activating pathways involved in tissue repair. Little is known about the regulation of HIF3α. Here we investigated the role of HIF3α *in vitro* and *in vivo*. Human MSCs expressed HIF3α, differentially regulated by pro-inflammatory cytokines in an oxygen-independent manner, a novel and still uncharacterized mechanism, where NF-κB is critical for its expression. We investigated if epigenetic modifications are involved in HIF3α expression by methylation-specific PCR and histone modifications. Robust hypermethylation of histone H3 was observed across HIF3A locus driven by pro-inflammatory cytokines. Experiments in a murine model of arteriotomy highlighted the activation of Hif3α expression in infiltrated inflammatory cells, suggesting a new role for Hif3α in inflammation *in vivo*.

## Introduction

Human mesenchymal stromal/stem cells (hMSCs) are multipotent cells able to differentiate along different mesenchymal lineages^1,2^. These cells have been proven as an attractive system in regenerative medicine for their therapeutic efficacy in various diseases, including heart attack, stroke and critical limb ischemia, resulting from their combined secretory, neoangiogenetic and immunomodulatory activities^3–5^. Several clinical trials are currently investigating autologous hMSC therapy in ischemic diseases with promising, but limited results, as only a small fraction of infused hMSCs homes in diseased tissues, where hypoxia and inflammation coexist.

Reduced oxygen availability triggers a cascade of pathophysiological events as adaptive mechanism to respond to hypoxic stress where hypoxia-inducible factors (HIFs) play a key role. HIFs are heterodimers consisting of a α subunit, whose expression is tightly regulated by oxygen availability, and a β subunit, constitutively expressed in all cells^6^. Three α subunits are present in mammals and most of our knowledge is based on HIF1α and HIF2α, sharing high sequence identity and regulation^7^. HIF1α and HIF2α protein abundance is regulated by two hydroxylation events in the oxygen-dependent degradation domain (ODD) by prolyl hydroxylases (PHD) and in C-terminal transactivation domain (C-TAD) by factor inhibiting HIF-1 (FIH). Under hypoxia, hydroxylation is reduced and HIF1α and HIF2α are accumulated. They dimerize with HIF1β, bind to specific sequences termed hypoxia response element (HRE) and activate a number of genes encoding proteins mainly involved in neoangiogenesis, e.g. vascular endothelial growth factor (VEGF), VEGF receptor-1 (VEGF-R1), stromal cell-derived factor-1 (SDF-1), and epidermal growth factor (EGF) and cell metabolism^8–11^.

In addition, hypoxia activates the expression of nuclear factor-kappa B (NF-κB), which in turn stimulates the release of pro-inflammatory cytokines, as interleukin 6 (IL6), tumour necrosis factor α (TNFα), and monocyte chemoattractant protein 1 (MCP1)^12^, revealing a functional relationship between hypoxia and inflammation.

Little is known about the third member of the family, HIF3α, sharing low sequence identity with the other two members^13^. In mammals, the HIF3A locus undergoes a complex regulation, giving rise to six splicing variants^14,15^, making difficult the elucidation of its function. None of them contains the C-TAD domain, while the ODD domain is highly conserved^16^. Hypoxic induction of HIF3α is regulated either at transcriptional level, differently from HIF1α and HIF2α, and at protein level, like the others^15^, *via* proteasomal degradation in a oxygen-dependent manner and also by microRNAs^17^, indicating a fine-tuning mechanism of HIF3α regulation. Furthermore, *in vitro* data in mammalian cells suggest that some HIF3α isoforms suppress HIF1α and HIF2α expression, while others inhibit HIF1α activity in a dominant negative fashion by competing with HIF1β^14,15,18,19^.

Gene targeting in mice suggests that NEPAS/Hif3αα plays a role when angiogenesis is required^20^, whereas, in zebrafish, it activates the inflammatory response in a oxygen-dependent manner^21^ and has some oxygen-insensitive role^22^.

Several oxygen-independent mechanisms also regulate HIF3α. Glucose deprivation, as well as insulin^23^, can increase the mRNA expression of some HIF3α isoforms^24^. In a genome-wide analysis of DNA methylation, HIF3α locus is silenced by hypermethylation in blood cells and in adipose tissue of adults with high body mass index (BMI), pointing to a role in metabolic response^25^.

Although informative, these studies did not clarify the function of human HIF3α gene, and our understanding of its regulation is still limited.

This prompted us to further investigate HIF3α in hMSCs, known to be endowed with secretory and immunosuppressive functions, *in vitro* and in rats infused with allogenic MSCs after carotid injury *in vivo*. Our findings provide new insights into the mechanisms of HIF3α regulation and its potential role in inflammation *in vitro* and *in vivo*, adding new elements in elucidating the mechanism of injury and repair and opening attempt to make therapy of ischemic disorders more efficient.

## Results

### HIF3α is regulated by cytokines in hMSCs in an oxygen-independent manner

Human mesenchymal stem cells (hMSCs) were cultured under standard oxygen conditions or hypoxic conditions for 24 hours and HIF3α protein was assessed by immunofluorescence.

Hypoxia was obtained either by Gas-Pak method, used to achieve 1% oxygen tension (H) or by the addition of cobalt chloride (CoCl_2_ 250 μM), a widely used hypoxia mimicker^26^. HIF1α expression, known to be expressed under hypoxia^7^, was used as positive control. HIF-1α was absent in standard oxygen conditions (N), but was nicely accumulated either in 1% O_2_ (H) or in CoCl_2_-induced hypoxia (CoCl_2_) (Suppl. Fig. 1a). To compare the effects of 1% O_2_ and CoCl_2_-induced hypoxia, we investigated also whether known hypoxia target genes were indeed activated. Expression analysis showed a significant induction of FLT1, GLUT1 and KDR mRNAs in hMSCs in 1% O_2_ (H) and in CoCl_2_-induced hypoxia (CoCl_2_) (Suppl. Fig. 1b), indicating that both methods (Gas-Pak vs. CoCl_2_) gave similar results in term of induction of hypoxia signalling, and therefore we used CoCl_2_-induced hypoxia for our experiments.

**Figure 1:**
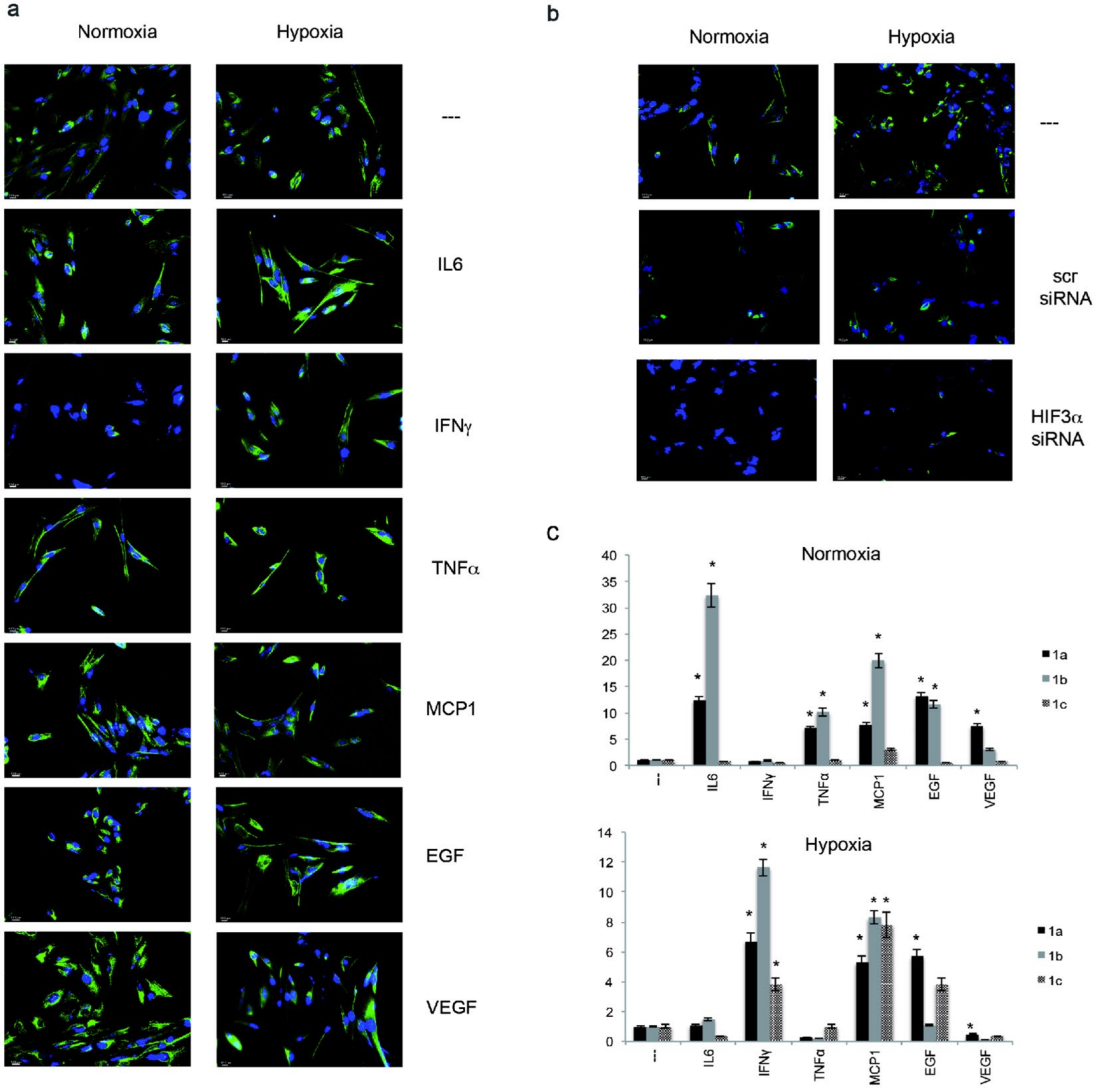
HIF3α expression in hMSCs. (**a**) Immunofluorescence analysis of HIF3α protein in hMSCs cultured in standard oxygen conditions (Normoxia), and in CoCl_2_-induced hypoxia (Hypoxia) for 24 h in absence and in presence of indicated cytokines and probed with antibodies against HIF3α. Scale bars: 10 μm). (**b**) Immunofluorescence analysis of HIF3α protein in cells grown in normoxia and hypoxia with siRNA-mediated HIF3α silencing or scrambled siRNA as control (scr). (**c**) Expression levels of the three alternative first exons (1a, 1b, and 1c) by qRT-PCR in hMSCs cultured in normoxia or hypoxia for 24 h in absence or in presence of indicated cytokines. Relative gene expression data are reported as 2-ΔΔCt method, normalized to housekeeping gene (b-actin mRNA) and ALU sequences. Data are expressed as means ± SEM (n = 3).*p value < 0.05.

In contrast to HIF1α, immunofluorescence (IF) analysis indicated that HIF3α was expressed in hMSCs cultured in normoxic conditions (21% O_2_) and it is predominantly localized to the cytoplasm of MSC cells (Fig. 1a). HIF3α was induced in CoCl_2_-induced hypoxic conditions and the protein remained into the cytoplasm, with a much weaker nuclear staining (Fig. 1a).

Since HIF proteins are subjected to oxygen-dependent and -independent mechanisms of regulation, we then explored whether HIF3α expression was influenced by a set of inflammatory cytokines. hMSCs were grown in presence of pro-inflammatory (IL6, IFNγ, TNFα, MCP1) and pro-angiogenic (EGF, VEGF) cytokines, and exposed to normoxia and CoCl_2_-induced hypoxia for 24 h. Given the dependence of hMSC proliferation on different cytokines, we first examined cell cycle under these conditions. As shown by flow cytometry analysis, none of the cytokines altered the G_1_ phase both in normoxia and in CoCl_2_-induced hypoxia (Suppl. Fig. 2a), although the presence of cytokines had a protective effect on apoptosis compared to control cells (Suppl. Fig. 2b).

IF staining further revealed that HIF3α expression was higher when hMSCs were cultured in presence of cytokines, both in normoxic and in CoCl_2_-induced hypoxic conditions (Fig. 1a). Remarkably, HIF3α was stimulated in presence of IL6, TNFα, MCP1, EGF and VEGF in normoxic conditions, whereas IFNγ had a negative effect. Furthermore, HIF3α showed similar staining pattern among cytokines in hypoxic conditions, in terms of quantity and localization.

As expected, HIF1α accumulation was only induced by CoCl_2_-induced hypoxia (Suppl. Fig. 2c).

To verify that the observed staining was HIF3α-specific, we used an RNA interference approach to silence HIF3α expression. hMSCs were transfected with a pool of HIF3α-specific siRNAs (ID 64344; NM_152795.3, exon 3–5) or with a scramble siRNA as negative control, and HIF3α expression was assessed by IF. A dramatic reduction of HIF3α expression was obtained both in normoxia and hypoxia, confirming the staining specificity (Fig. 1b).

We then looked at HIF3α mRNA expression. Six splicing isoforms with three alternative start sites were identified with different expression patterns in foetal and adult tissues^14,15^. We evaluated the expression levels of the three alternative exons of HIF3α mRNAs (exon 1a for isoforms 2, 4 and 9; exon 1b for isoforms 7 and 8; exon 1c for isoforms 1, Suppl. Fig. 3a) by qRT-PCR^15^. The expression of HIF3α mRNAs, starting from exon 1a and exon 1b, was greatly induced when hMSCs were exposed to cytokines in standard oxygen conditions (N), except IFNγ, paralleling the IF data, whereas expression isoform starting from exon 1c was barely induced (Fig. 1c). In CoCl_2_-induced hypoxia, mRNAs starting from exon 1a, 1b and 1c were further induced only in presence of IFNγ, MCP1 and EGF (Fig. 1c).

Taken together, these data suggested that hMSCs expressed HIF3α in standard oxygen conditions, but, more important, the endogenous level of HIF3α is induced by pro-inflammatory cytokines in a oxygen-independent manner and it is localized to the cytoplasm of hMSCs.

### HIF3α is regulated by NF-kB in a oxygen-independent manner

Given that HIF3α showed an increased expression in presence of inflammatory cytokines independent from oxygen, we investigated whether NF-κB could be implicated in its activation^27^.

**Figure 2:**
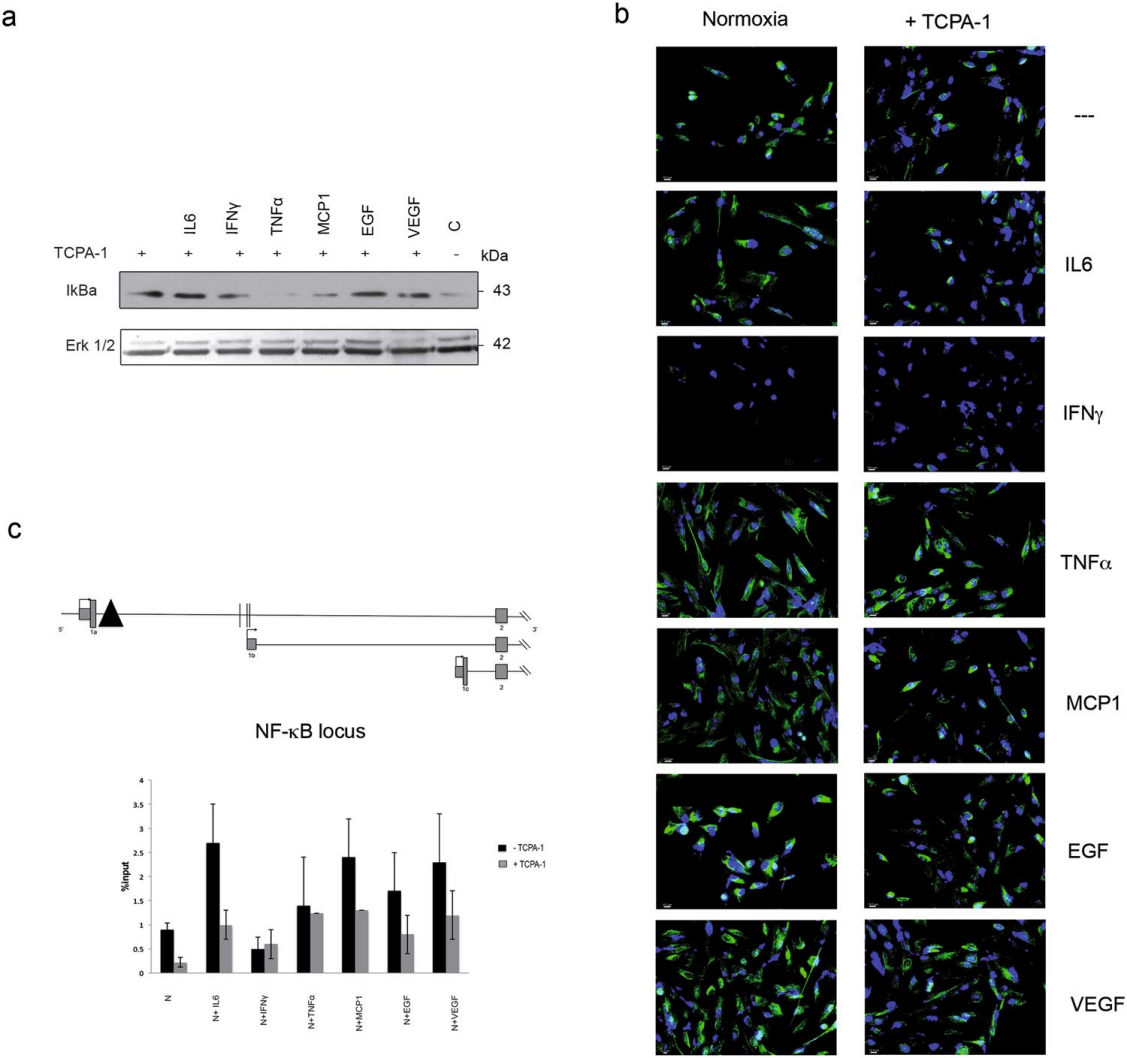
HIF3α activation is dependent on NF-κB activation: (**a**) Immunoblotting analysis of IκBα: hMSCs cultured in normoxia and treated with IL6, IFNγ, TNFα, MCP1, EGF and VEGF for 24 h. Cells were pre-treated with TCPA-1 for 1 h before cytokines supplementation. (**b**) Immunofluorescence analysis of HIF3α protein in hMSCs cultured in standard oxygen conditions (Normoxia) in absence and in presence of indicated cytokines and probed with antibodies against HIF3α. Cells were pre-treated with TCPA-1 for 1 h before cytokines supplementation. Scale bars: 10 μm). (**c**) Schematic diagram of the HIF3A promoter region, where a putative NF-KB binding site is depicted (black triangle). NFκB (RelA) binding on HIF3A promoter: ChIP analysis was performed in hMSCs cultured in standard oxygen conditions and treated with IL6, IFNγ, TNFα, MCP1, EGF and VEGF for 24 h (black bars), or pre-treated with TCPA-1 for 1 h (grey bars) before adding cytokines. As control, species matched IgG were used. Data obtained by qRT-PCR are expressed as enrichment of chromatin-associated DNA fragments immunoprecipitated by NF-κB antibody compared with input (% Input) and expressed as means ± SEM of two independent experiments performed in triplicate.

To test this, we treated hMSCs with TCPA-1^28,29^, a compound with the potential to inhibit NF-κB by preventing the degradation of IκBα. We first assessed the IκBα expression in hMSCs grown under normoxic conditions in presence of TCPA-1. As shown in Fig. 2a, hMSCs accumulated IκBα when cultured in presence of cytokines in standard oxygen conditions in presence of TCPA-1, compared to untreated cells, with the exception of TNFα (Fig. 2a). In these conditions, IF analysis revealed that the expression of HIF3α was reduced by TCPA-1, indicating that the cytokines became ineffective in inducing HIF3α (Fig. 2b). In contrast, in CoCl_2_-induced hypoxia, IκBα was not accumulated (Suppl. Fig. 4a), and no effects were seen on HIF3α expression by IF (Suppl. Fig. 4b), probably because hypoxia signalling was prevalent.

Thus, NF-κB is seemingly involved in HIF3α activation under standard oxygen conditions.

NF-κB binds a large number of genes activated by inflammation in the human genome^30^. Then, we searched for κB sites in promoter sequence of HIF3A using JASPAR software^31^ and we found in intron 1a a putative κB binding site (Fig. 2c). Chromatin immunoprecipitation with a specific NF-κB (RelA) antibodies was performed in hMSCs cultured in standard oxygen conditions and stimulated by indicated cytokines. After cytokines stimulation, 2- to 3- fold enrichment of NF-κB binding in this region was observed and the pre-treatment of cells with TCPA-1 reduced this binding, suggesting that HIF3α expression in hMSCs is induced by cytokines via NF-κB (Fig. 2c).

### HIF3α expression correlates with promoter methylation and histone modifications

**Figure 3:**
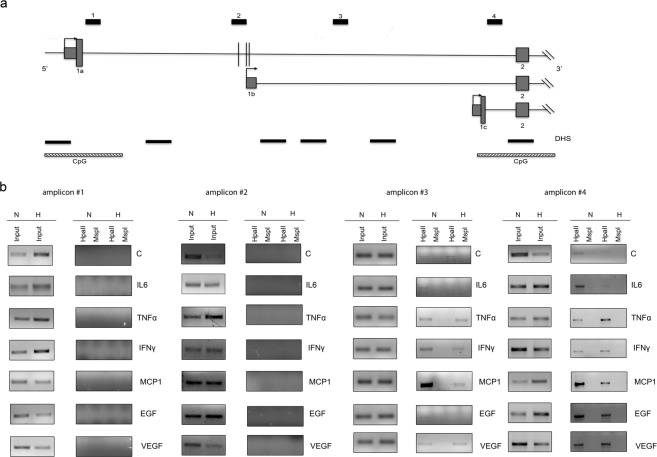
DNA methylation status of human HIF3A promoter. (**a**) Schematic diagram of the HIF3A promoter region, CpG islands and DNAse I hypersensitive sites (DHS) surrounding the three transcription start sites. Vertical black lines represent the three methylation sites in intron 1 in association with high BMI^25^. (**b**) MS-PCR analysis of genomic DNA extracted from hMSCs exposed to different pro-inflammatory cytokines in normoxic and hypoxic conditions. The locations of PCR amplicons (#1 to #4) are shown in (**a**). Three independent experiments were performed and a representative experiment is reported. Images derived from different part of the same gel and cropped for layout reasons and included in Suppl. Information.

To gain further insights on HIF3A gene regulation, we examined the methylation status of the three different promoters. We selected regions by the presence of restriction enzyme sites sensitive to methylation (HpaII and MspI). Two CpG islands, defined as DNA sequences with at least 50% GC content over a minimum of 300 bp, were identified on human HIF3A gene, overlapping exon 1a and exon 1c. Our analysis encompassed also the regions identified by high-density DNA methylation array experiments and covering the exon 1b and the intron 1, as depicted in Fig. 3a, and found hypermethylated^25^. Genomic DNA was extracted from hMSCs exposed to pro-inflammatory cytokines in normoxic and CoCl_2_-induced hypoxic conditions and subjected to methylation-sensitive-restriction enzyme PCR (MS-PCR) analysis^32^. Amplicons #1 and #2 were efficiently digested both by HpaII and MspI enzymes in presence of cytokines under normoxic and CoCl_2_-induced hypoxic conditions, indicating the absence of any DNA methylation in these regions (Fig. 3b). In contrast, a peculiar restriction pattern was seen over the intron 1 (amplicon #3), which was methylated in hMSCs grown either in normoxia and in CoCl_2_-induced hypoxia and resulted unmethylated in presence of IL6, IFNγ and EGF in hypoxic conditions, indicating that this modification is reversible. Loss of methylation in this site suggested that pro-inflammatory cytokines are able to activate the expression of HIF3α isoforms starting from exon 1b. In contrast, the region covering exon 1c (amplicon #4) showed cleavage resistance to HpaII in DNA extracted from hMSCs grown in all conditions, indicating a persistent DNA methylation and suggesting that the HIF3α isoform starting from exon 1c is barely transcribed in hMSCs.

**Figure 4:**
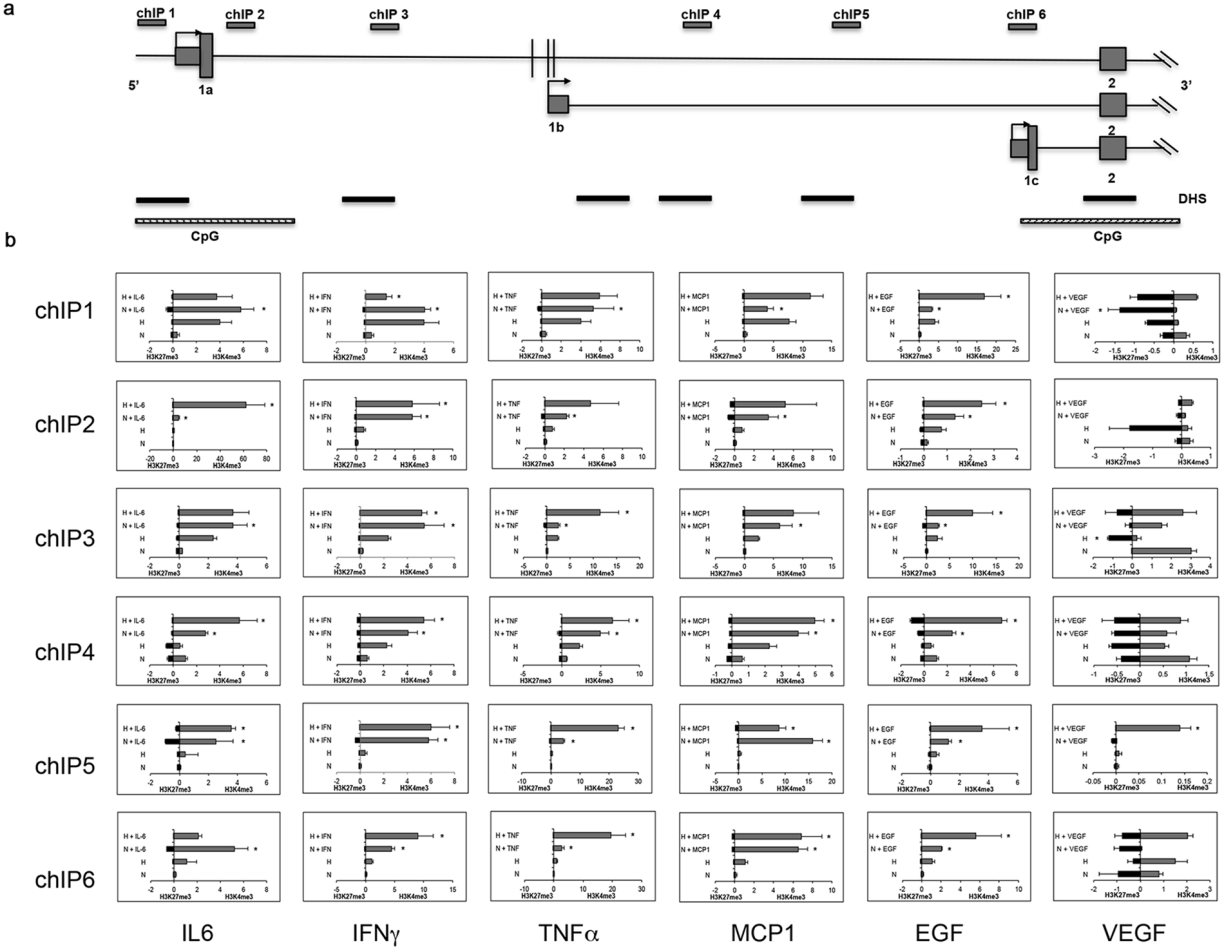
ChIP-PCR analysis of histone modifications of human HIF3A promoter. (**a**) Schematic diagram of the HIF3A promoter region and primers used in ChIP analysis using specific antibodies to H3K4me3 and H3K27me3 histone modifications. qRT-PCR was performed on immunoprecipitated chromatin of hMSCs treated with different cytokines in normoxic and hypoxic conditions. (**b**) Six different regions (ChIP1-6) spanning the HIF3A gene were analysed. Results are expressed as means ± SEM of three different experiments and are reported as the ratio between treated hMSCs *vs*. untreated hMSCs (2-ΔΔCt method). *< 0.05 (p-value).

Next, we verified whether pro-inflammatory cytokines and/or CoCl_2_-induced hypoxia might induce changes in the chromatin structure around the HIF3α transcription start sites. Chromatin immunoprecipitation assay (ChIP) was performed using specific antibodies against trimethylation of lysine 4 in histone H3 (H3K4me3), mark of active transcription, and trimethylation of lysine 27 in histone H3 (H3K27me3), associated with gene silencing. Six different regions spanning HIF3α gene were analysed on the basis of currently available methylome data (Fig. 4a). When hMSCs were exposed to cytokines and cultured at standard and low oxygen tension, we found a global enrichment of H3K4me3 marks on all regions analysed and low H3K27me3 levels, compared to untreated hMSCs (Fig. 4b).

Notably, an enrichment of H3K27me3 was observed when hMSCs were exposed to VEGF both in normoxia and hypoxia across the region chIP1, 2 4 and 5, possibly indicating that these regions are paused and might be activated by additional signals. (Fig. 4b).

In conclusion, our data support the hypothesis that the HIF3α locus is sensitive to inflammatory cytokines that are able to modify the epigenetic profile independent from oxygen conditions.

### Immunohistochemical analysis of Hif3α in a murine model of arteriotomy

**Figure 5:**
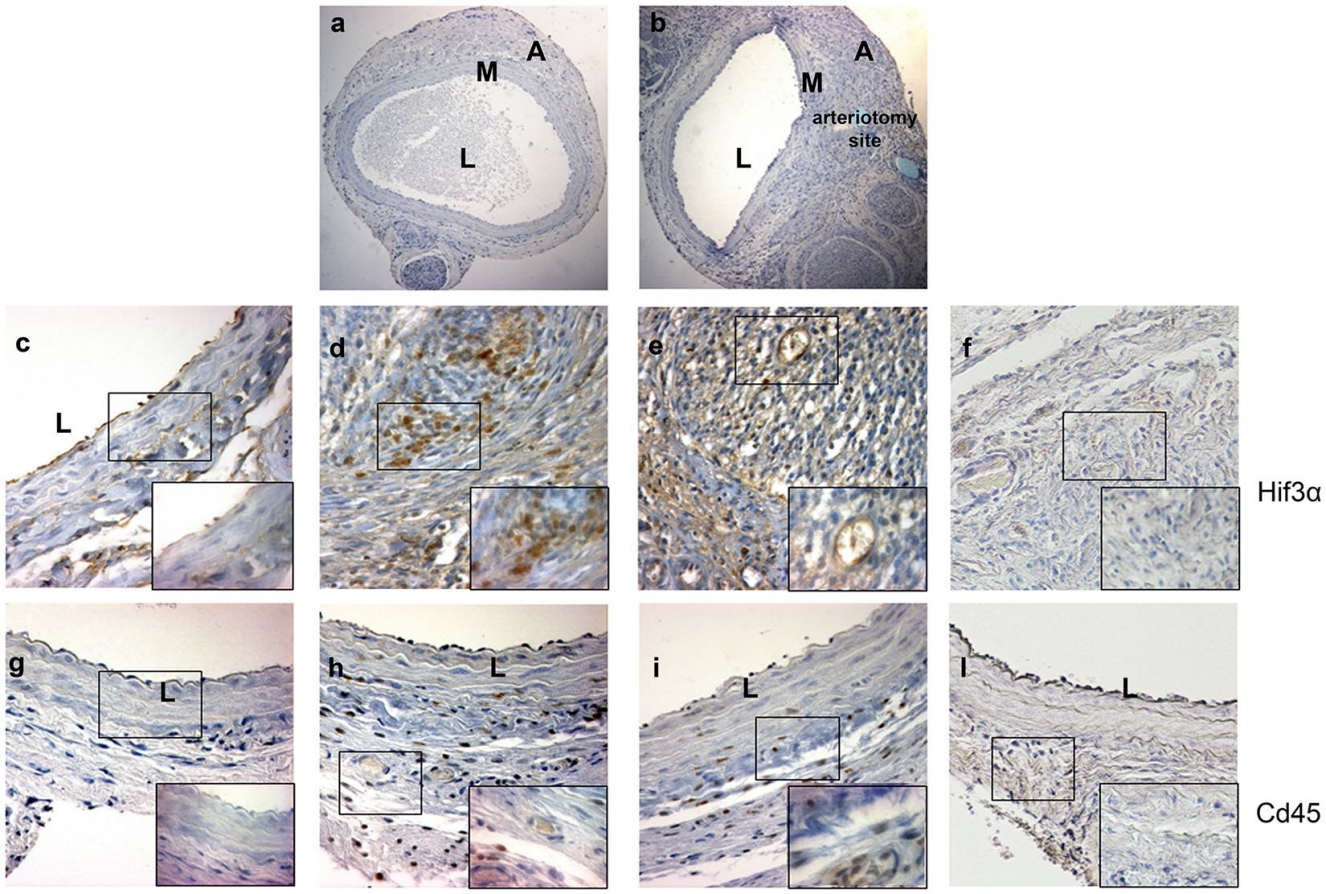
Immunohistochemical analysis of Hif3α and Cd45 expression in uninjured rat carotids and in carotids from Wistar male rats treated with MSCs or DMEM and harvested 7 days after arteriotomy. (**a**) Uninjured rat carotid; (**b**) arteriotomy-injured rat carotid harvested 7 days after injury, haematoxylin staining. Arrows in b indicate the injury site, where arteriotomy is followed by the application of an 8.0 polypropylene stitch (light blue). (**c**–**f**) Representative immunohistochemical staining of Hif3α in uninjured rat carotid (**c**) and in injured carotids harvested 7 days after arteriotomy from DMEM- (**d**) and MSC-treated rats (**e**). (**g**–**l**) Representative immunohistochemical staining of Cd45 in adjacent cross-sections from uninjured rat carotid (**g**) and in injured carotids harvested 7 days after arteriotomy from DMEM- (**h**) and MSC-treated rats (**i**). (**f**,**l**) Immunohistochemical staining of serial cross-sections of rat carotids used in d and h without primary antibody as negative control. (**a**,**b**) 10x magnification; (**c**–**l**) 20x magnification; small insets: 40x magnification of selected areas enclosed in black rectangles, representative of nuclei positive to Hif3α and Cd45 in adventitia, *vasa vasorum* and perivascular tissue. Brown staining corresponds to target protein expression. Nuclei were counterstained with haematoxylin. L: lumen; M: media; A: adventitia.

We then investigated the relevance of the *in vitro* data to an acute pathophysiological condition *in vivo*. In more detail, Hif3α activation was assessed in a well-established model of rat carotid arteriotomy, in which vascular damage triggers a cascade of events, where inflammation and hypoxia coexist^33^. Rat common carotid arteries were subjected to surgical arteriotomy and animals were administered *via* tail vein with allogenic MSCs or with DMEM soon after the vascular injury. Our previous studies demonstrated the efficacy of MSC administration in limiting the inflammation triggered by arteriotomy^5^, and consequently this model of acute injury has been considered advantageous and potentially informative in the setting of Hif3α research. Arteriotomy-injured rat carotids were harvested 7 days after injury. Injured carotids exhibited a decreased lumen area (Fig. 5b) compared to controls (Fig. 5a), resulting mainly from negative remodelling and thickening of neoadventitia close to the polypropylene stitch, causing a lumen reduction due to *ab-estrinseco* compression of the artery^33^. The induction of hypoxia at the arteriotomy site was verified by HIF1α expression (Suppl. Fig. 5c–e). Immunohistochemical analysis (IHC) revealed that HIF1α was expressed only in sporadic endothelial cells at basal level in uninjured carotids, possibly due to hypoxia occurring during the surgical procedure for carotid removal (Suppl. Fig. 5c). Conversely, HIF1α expression was markedly increased 7 days after injury in the carotid region proximal to the arteriotomy site, with particular reference to infiltrating cells in the adventitia, and to intimal and adventitial endothelial cells in *vasa vasorum* (Suppl. Fig. 5e). In the region distal to the arteriotomy site within the same carotid cross-section, adventitial *vasa vasorum* and infiltrating cells were negative and HIF1α expression was limited to sporadic endothelial and smooth muscle cells (Suppl. Fig. 5d).

We then carried out IHC analysis to assess whether neoadventia relied also on infiltration of inflammatory cells. Cd45, a general marker of leukocytes, is expressed in infiltrating round-shaped cells in neoadventitia (infiltrated leukocytes), and in fibrocyte-like cells in outer media of injured carotids (Fig. 5h). On adjacent carotid cross-sections, Hif3α was markedly expressed in the same round-shaped cells in neoadventitia tissue (Fig. 5d), suggesting a consistent activation of this factor in inflammatory cells. The absence of staining in negative controls of immunohistochemical reactions confirmed the specificity of the assay (Fig. 5f and l), suggesting that Hif3α expression is inducible upon inflammation.

The same IHC analysis performed in MSC-treated rats submitted to carotid arteriotomy confirmed Hif3α activation in infiltrating cells (Fig. 5e), suggesting that the MSC immunomodulatory role did not inhibit Hif3α expression. Noticeable, Hif3α was expressed also in perivascular cells and in adventitial endothelial cells in *vasa vasorum* of arteriotomy-injured carotids in MSC-treated rats (Fig. 5e, small inset), suggesting its possible involvement in tissue damage recovery, whereas it was absent in adventitial endothelial cells in *vasa vasorum* of arteriotomy-injured carotids in DMEM-treated rats (Fig. 5d). All together, our data showed that Hif3α is expressed in inflamed tissue and might contribute to the repair process.

## Discussion

Low oxygen concentration within a tissue activates a compensatory mechanism to counteract the detrimental effects of lack of oxygen and nutrients. Concomitantly, it induces an inflammatory status, and several studies have pointed to inflammation as an essential process to activate the repair process during which inflammatory cells infiltrate the hypoxic tissue, produce different cytokines to sustain inflammation, and activate neoangiogenesis and cell metabolism^27^. HIFs and NF-κB play a crucial role in this mechanism, activating the expression of genes involved in neoangiogenesis^9–11,34^, and pro-inflammatory factors^35^, indicating a tight biochemical and functional relationship between hypoxia and inflammation. Our data indicate a role for HIF3α in the early response to inflammation, and activate neoangiogenesis to repair the damaged tissue.

Up to now, little is known about HIF3α. This is in part due to the existence of multiple isoforms; according to Ensembl database, the human Hif3A has 19 transcripts in total, of which only 6 experimentally confirmed with different functions. Some HIF3α isoforms suppress HIF1α and HIF2α expression, while others inhibit HIF1α activity in a dominant negative fashion^14,15,18,19^. Recent transcriptomic data in zebrafish suggested that Hif3α activates the inflammatory response^21^.

In this study, we report for the first time that human HIF3α expression is modulated by pro-inflammatory cytokines *in vitro*, and is associated with inflammation and neoangiogenesis *in vivo*, providing a direct involvement of HIF3α in repair process.

Multiple lines of *in vitro* evidence support our conclusions: (a) IF data showed that HIF3α increases when hMSCs are cultured in standard oxygen conditions and exposed to pro-inflammatory cytokines; (b) Hypoxia further induced HIF3α accumulation, as for other HIF family members. Moreover, IF staining showed a peculiar cytoplasmic localization for HIF3α, a protein supposed to be a transcription factor; (c) HIF3α accumulation is regulated by NF-κB in oxygen-independent manner: hMSC treatment with TCPA-1, a IκB inhibitor able to sequester NF-κB in the cytoplasm, reduces HIF3α activation; (d) chromatin immunoprecipitation reveals that NF-κB binds the HIF3A intronic region and pre-treatment with TCPA-1 reduces its binding; (e) exposure of hMSCs to cytokines modifies the methylation status of HIF3A promoter regions with a subsequent enrichment of the H3K4me3 marks, indicating that cytokines are able to open the HIF3A locus; (f) in a murine model of carotid arteriotomy, Hif3α is activated *in vivo* and is expressed in infiltrating inflammatory cells of damaged tissue and in *vasa vasorum* endothelial cells in repairing process. Furthermore, Hif3α protein localisation was slightly different from Hif1α in arteriotomy-injured carotids, with a prevalence of Hif3α expression in adventitia, whereas Hif1α is mainly accumulated in the intima, reflecting a gradient of expression between the two factors.

The new findings suggest that HIF3α gene is sensitive to inflammatory cytokines and is involved not only in inflammation, but also in neoangiogenesis associated with injury resolution and tissue repair. Infiltration of inflammatory cells producing proangiogenic factors is one of first event that then stimulate neoangiogenesis in the context of inflamed and hypoxic tissue. Our data showed that HIF3α is expressed in the infiltrating inflammatory cells, suggesting the possibility that it functions as sensor of inflammation/hypoxia in injured tissue.

## Conclusions

Inflammation and neoangiogenesis are both involve in tissue repair. Our data revealed that human HIF3α expression is regulated by inflammatory cytokines in hMSCs in a oxygen-independent manner, showing a distinct mechanism of regulation, compared to HIF1α. Furthermore, the expression is in part under the control of NF-κB. In addition, to the best of our knowledge, the expression of Hif3α in both inflammatory and endothelial cells has been revealed for the first time *in vivo* in a murine model of vascular restenosis, suggesting that Hif3α might play a role in the activation of tissue repair and its acceleration in presence of allogenic MSCs, endowed with immunomodulatory and secretory properties.

Despite the progress, a lot of questions remain open. The human Hif3A has 19 transcripts in total, of which only 6 experimentally validated in different mammalian cells, including cancer cells. Have these isoforms a cell-context function? What is the role of HIF3α in immune cells? Is HIF3α important for the interaction of different immunomodulatory cells? In humans with high BMI, often associated with metabolic syndrome characterized by chronic inflammation, is HIF3α silenced pointing out a role in metabolic surveillance? In cancer cells, is HIF3α involved in neoangiogenesis to sustain tumour growth? or induces metastasis by altering the cytoskeleton of cancer cells^36^?

Additional studies will be necessary to clarify the exact role of HIF3α in different normal and cancer cells and in different growth conditions; the newly CRISPR/CAS technology will help us to better define its role.

We believe that any progress toward the comprehension of this progress will have important clinical implications for treatment of ischemic disorders and cancer.

## Methods

### hMSC culture and treatment

hMSCs were obtained and characterized, as previously described^37^. Cells were grown in RPMI-1640 medium (Euroclone SPA, Italy), containing 10% heat-inactivated foetal bovine serum (FBS), 1% Penicillin-streptomycin, and 1% L-Glutamine, and maintained as monolayers in a humidified atmosphere containing 5% CO_2_ at 37 °C. Hypoxic culture conditions were achieved in a BD GasPak EZ Anaerobe Gas Generating Pouch System (BD Biosciences, San Diego). As certified by the manufacturer, the Anaerobe Gas Generating Pouch System produces an atmosphere containing 1% oxygen after 1 h. Hypoxic culture conditions were also obtained adding 250 μM CoCl_2_ (Sigma-Aldrich, Saint Louis, MO, USA), a hypoxia mimetic agent, in culturing medium.

hMSCs were treated with different concentrations of cytokines for 24 h in normoxic and hypoxic conditions. Cytokines used in the study were: EGF and MCP1 (5 ng/mL and 20 ng/mL, respectively, Provitro, Berlin, Germany), VEGF, TNFα, IL6, IFNγ (25 ng/mL, 10 ng/mL, 20 ng/mL and 100 ng/mL, respectively ISOkine, ORF genetics, Kopavogur, Iceland). For TCPA-1 (5μM, a gift of Dr. Ursini, Sigma-Aldrich, Saint Louis, MO, USA)^28,29^, the drug was added to culture medium one hour before cytokines addition.

### Silencing experiments

hMSCs were seeded into 12-well plates at 5 × 10^4^ cells/well density. After 24 h, hMSCs were transfected with 50 nM of small interfering RNAs (siRNAs) specific for exon 3–5 of human HIF3A (ID 64344, Riboxx, Life Sciences, DE) using Lipofectamine 2000 (Life Technologies, USA), according to the manufacturer’s protocol. The day after transfection, cytokines were added, as described. After 48 h, immunofluorescence was performed.

### RNA preparation and qRT-PCR

Total RNA was isolated from hMSCs by miRNeasy Mini kit (Qiagen, Hilden, Germany) and 500 ng were converted to cDNA by reverse-transcription using Quantitect RT Kit (Qiagen). qRT-PCR was performed using SybrGreen PCR Master mix 2x reagent in the CFX96TM Real Time PCR Detection Systems (BioRad, CA, USA). HIF3α mRNA expression was normalized using the β-actin gene as housekeeping gene and ALU repeats^38^.

### Protein extraction and Western blot analysis

hMSCs were incubated with different concentrations of cytokines (25 ng/mL EGF, 25 ng/mL VEGF, 20 ng/mL IL6, 10 ng/mL TNFα, 100 ng/mL IFNγ, 20 ng/mL MCP1) for 24 h in normoxic and hypoxic conditions and cells were then lysated in 20 mM Tris-HCl, 100 mM NaCl, 10 mM MgCl_2_, 1% NP-40, 10% glycerol, 0.1 M NaF, 100 μM Na_3_VO_4_, and protease inhibitors mixture (Roche Ltd, Basel, Switzerland). Equal amounts of proteins were separated by SDS-polyacrylamide gels and transferred to nitrocellulose membranes (Whatman, GE Healthcare, Europe). Membranes were incubated with blocking buffer (TBS-Tween buffer containing 5% milk) for 1 h at room temperature and subsequently with primary antibodies at 4 °C overnight. After three washes for 10 min with TTBS buffer (50 mM Tris-HCl, pH 8.0, 150 mM NaCl, and 0.5% Tween-20), the membranes were incubated with horseradish peroxidase-conjugated anti-rabbit antibody (1:10.000, Amersham, GE Healthcare, Life Technologies, Europe) for 1 h at room temperature and then washed for 15 min with TTBS buffer. The resulting immunoblots were detected using Amersham ECL Plus (GE Healthcare, Life Technologies, Europe).

### Antibodies and oligonucleotides

The following antibodies were used in this manuscript:

Rabbit polyclonal anti HIF1alpha (H-206, sc-10790, Santa Cruz Biotechnology, CA, USA) for western blotting and IHC, rabbit polyclonal anti-HIF3 alpha (orb215263, Biorbyt Ldt, UK) for immunofluorescence, rabbit polyclonal anti-HIF3 alpha (sc-28707, Santa Cruz Biotechnology) for IHC, rabbit monoclonal anti-NF-kappaB (D14E12, Cell Signalling Technology, Europe) and mouse monoclonal anti-CD45RO (UCHL-1) (Ventana Medical Systems Inc., USA) for IHC. Mouse monoclonal antibodies directed against beta tubulin (D3U1W, Cell Signalling Technology, Europe) and ERK1/2 (7D8, Abcam) were used for western blotting as loading control.

Furthermore, we also used goat polyclonal anti-HIF3alpha antibody (sc#32144, C-18, SantaCruz Biotech.), rabbit polyclonal anti-HIF3alpha antibody (sc#32142, T-15, SantaCruz Biotech.), mouse monoclonal antibodies (sc#390933, E-8, SantaCruz Biotech.) for western blotting analysis in human MSC extracts with no results.

Rabbit polyclonal anti-HIF3alpha antibody (sc#28707x, H170, SantaCruz Biotech.) and mouse monoclonal anti-HIF3alpha [OTI2D2] (ab139280, Abcam discontinued, TA800720 Origene) for western blotting and immunoprecipitation analysis with no results.

For chIP experiments, H3K4me3 (C15410003 - Diagenode), H3K27me3 (C15200181- Diagenode) and NF-κB (D14E12, Cell Signalling Technology, Europe) antibodies were used. Related IgG were purchased by Santa Cruz Technology.

Oligonucleotides used in this study are reported in Table 1.

**Table 1:** Primers sequence from 5′ to 3′ used in this study.

Oligo Name	Sequence (5′->3′)
**MSR-PCR:**	
HIF-3α amplicon Fw #1	CCGCCCCCATCCTCTCCCC
HIF-3α amplicon Rw #1	CCATCGCCCAGGCCCCCG
HIF-3α amplicon Fw #2	CCTGGAGACCCCTGAGCTGGATTGT
HIF-3α amplicon Rw #2	CCCGCAGAAGCCTGGGGACTGCTCA
HIF-3α amplicon Fw #3	CGGATTCACTGAGAAGTGGTTG
HIF-3α amplicon Rw #3	CTCACGGAGCTAGAGAACCAT
HIF-3α amplicon Fw #4	ACTGCAGATAAGTCAGGGAGGG
HIF-3α amplicon Rw #4	AAAGAGAAAAGGAGGACGGGAC
**ChIP qPCR:**	
HIF-3α ChIP Fw #1	GCTCAACTGGGGTTAGGAAATG
HIF-3α ChIP Rw #1	ATGTCACTCCTGAAAAGGAGGC
HIF-3α ChIP Fw #2	CCTGGAGACCCCTGAGCTGGATTGT
HIF-3α ChIP Rw #2	CCCGCAGAAGCCTGGGGACTGCTCA
HIF-3α ChIP Fw #3	TCCAAGCTTTATTTTGGGGAGA
HIF-3α ChIP Rw #3	GACAGGGAAAGCTGAGGACCTA
HIF-3α ChIP Fw #4	CTGGGTATCACACTCCCTTTCC
HIF-3α ChIP Rw #4	CTCACGGAGCTAGAGAACCCAT
HIF-3α ChIP Fw #5	AGTAGAACATCCAGAGGGCAGG
HIF-3α ChIP Rw #5	TCTTCAGGCTTTTTCTCATCCC
HIF-3α ChIP Fw #6	ACTGCAGATAAGTCAGGGAGGG
HIF-3α ChIP Rw #6	AAAGAGAAAAGGAGGACGGGAC
**RT-qPCR:**	
HIF-3α Ex 1a Fw	GACTGGCGAGCCATGGCG
HIF-3α Ex 1b Fw	GTGCGCACCCACTCGTAACTCG
HIF-3α1 Ex 1c Fw	CGCCACAGAGAGGAGCGAGG
HIF-3α Ex 2 Rw	CACCTGGACAAGGCCTCTAT

### Immunofluorescence analysis

Cells were seeded the day before and grown on coverslips in 12-well plates^5^. After desired treatment, cells were fixed in Methanol (10 min, −20C), then blocked with BSA 2% in TBST for 1 h and stained with anti-HIF3a antibodies (1:100, orb215263, Biorbyt Ldt, UK) diluted in PBS-BSA overnight. The next day, the slides were washed three times with PBS-BSA and incubated with DyLight 488 conjugate anti-rabbit secondary antibodies (ImmunoReagents, Inc, Rb-003-D488 NHSX, 1:400) for 1 h at room temperature. Nuclei were counterstained with DAPI (10 μg/ml, Molecular Probes, D1306), extensively washed with PBS-BSA and mounted in Vectashield mounting medium (Vector Laboratories, H-1000, Burlingame, CA). Images were taken with Leica Microscope.

### Flow cytometry analysis

Cell cycle distribution was assessed with a FACScalibur flow cytometer (Becton Dickinson, San Jose, CA, USA), and 10,000 events were considered and analysed by ModFit version 3 (Verity Software House, Topsham, ME, USA) and Cell Quest (Becton Dickinson)^37^. hMSCs were treated with different concentrations of cytokines as described above. hMSCs were collected and then suspended in a in PBS 1x staining solution containing RNAse A, propidium iodide (50 μg/mL), sodium citrate (0.1%), and NP-40 (0.1%) for 30 min in the dark before cytometry analysis.

### MS-PCR

The DNA methylation analysis has been performed through the methylation sensitive restriction enzyme-polymerase chain reaction (MS-PCR) assay, a well-established method already applied in different settings^32,39^. Genomic DNA was extracted by lysing cultured hMSCs with 1% SDS followed by proteinase K digestion, ethanol precipitation, and phenol-chloroform purification. MS-PCR was performed on purified genomic DNA (1 μg) that was previously restriction endonuclease-digested for 48 h with the isoschizomers MspI and HpaII (New England Biolabs, USA).

### ChIP-qRT-PCR

Chromatin immunoprecipitation was performed as reported^30^. hMSCs were chemically cross-linked with 11% formaldehyde solution for 10 min at 37 °C. After a glycine/PBS wash, hMSCs were lysed with 0.1% SDS, 0.5% Triton X-100, 20 mM Tris–HCl (pH 8.0), 150 mM NaCl, and protease inhibitors, and sonicated on ice using pulses of 30 s separated by pauses of 30 s five times at maximum. Fragments derived from sonication of hMSC genomic DNA ranged from 200 bp to 500 bp in size, as verified through agarose gel electrophoresis (data not shown). The resulting whole-cell extract was incubated with anti-H3K27me3 and anti-H3K4me3 antibodies (code # C15410195 and code # C1541030, Diagenode, USA) overnight at 4 °C. The immunocomplexes were collected on Protein G-Sepharose, washed twice with lysis buffer, with washing buffer (0.1% SDS, 0.5% Triton X-100, 2 mM EDTA pH 8.0, 20 mM Tris–HCl pH 8.0, 150 mM NaCl, and protease inhibitors), once with LiCl buffer (0.25 mM LiCl, 1% NP-40, 1% sodium deoxycholate, 1 mM EDTA pH 8.0, 10 mM Tris–HCl pH 8.0, and protease inhibitors). Bound immunocomplexes were released with the elution buffer (1% SDS, 0.1 M NaHCO_3_ pH 8.0), 10 μl of 5 M NaCl added per sample and reverse cross-linked overnight at 65 °C. Genomic DNA was purified by multiple phenol:chloroform:isoamylic alcohol (25:24:1) extractions. Purified DNA was used as a template for qRT-PCR to amplify the proximal promoter regions of HIF3α. Primers used are listed in Table 1. The relative sample enrichment was calculated with the following formula: 2^ΔCtx-2^ΔCty, where x represents Ct input-Ct sample and y represents Ct input-Ct control Ab. Data shown are means of three independent experiments.

### Rat MSC culture

MSCs have been harvested from the bone marrow of the femurs and tibias of adult male Wistar rats, as reported^5,40^. Rats have been anaesthetized with an intraperitoneal injection of ketamine hydrochloride and MSCs have been harvested from the bone marrow by inserting a 21-gauge needle into the shaft of the bone and flushing it with complete α-modified Eagle’s medium (αMEM) containing 20% foetal bovine serum (FBS), 2 mM L-glutamine, 100 U/ml penicillin, 100 µg/ml streptomycin. Cells from one rat have been plated into two 100 mm dishes. After 24 hrs, non-adherent cells have been discarded, and adherent cells have been washed twice with PBS. The cells have been then incubated for 5–7 days to reach confluence. In order to obtain the sufficient amount of cells to be injected in rats at the time of arteriotomy, MSCs were cultured for 23 days, including 15 days from passage zero (corresponding to passage 5). All the cell culture reagents have been obtained from Invitrogen (Milan, Italy). Differentiation ability and senescence in cultured rat MSCs have been assessed, as previously published^40^.

### Rat carotid arteriotomy and MSC treatment

Arteriotomy of rat common carotid artery was performed as reported^33^. Male Wistar rats (Charles River, France) were anesthetized with i.p. injection of 100 mg/kg ketamine and 0.25 mg/kg medetomidine, and carefully placed onto a warm surface and positioned for surgery. All the surgical procedures were conducted under sterile conditions and vital signs were continuously monitored by pulsioxymeter. A plastic Scanlom clamp for coronary artery grafting was placed for 10 s on the carotid causing a crushing lesion to the vessel. At the same point where the clamp was applied, a 0.5 mm longitudinal incision was made on the full thickness of the carotid. The incision did not cross to the other side of the vessel. Haemostasis was obtained with a single adventitial 8.0-gauge polypropylene stitch. Once bleeding stopped, the carotid artery was carefully examined and blood pulsation was checked distally to the incision. A reabsorbable suture approximated the skin. Animals were allowed to wake up through an intramuscular injection of 1 mg/kg atipamezol. Postoperative systemic analgesia was administered through subcutaneous injection of 0.1 mg/kg buprenorphine every 8 h. Antibiotic therapy was administered through subcutaneous injection of 5 mg/kg enrofloxacin once a day for 3 days following the arteriotomy procedure. Rats submitted to arteriotomy were administered with 5 × 10^6^ MSCs suspended in 200 μl DMEM via tail vein injection (n = 5) soon after arteriotomy, while control rats were administered with 200 μl DMEM (n = 5).

### Immunohistochemistry

Immunohistochemistry was carried out as reported^5^. Carotid arteries were harvested 7 days after arteriotomy from MSC-treated rats (n = 5) and from DMEM-treated rats (n = 5). Control carotids were harvested from uninjured rats (n = 5). Harvested vessels were fixed in 4% buffered formaldehyde, dehydrated and embedded in paraffin. Consecutive 4% formaldehyde-fixed 5 μm cross-sections were deparaffinised and rehydrated. Antigen retrieval was performed in a microwave through incubation in 10 mM citrate buffer pH 6.0. Endogenous peroxidases were blocked with 4% H_2_O_2_. Blocking was carried out in 5% donkey serum, followed by incubation with the primary HIF3α antibody (H-170), with the HIF1α antibody (H-206) (Santa Cruz Biotech.) or with CONFIRM anti-CD45RO (UCHL-1) primary antibody (Ventana Medical Systems Inc., USA) at 1:100 dilution at 4 °C overnight. Immunostaining was performed manually (HIF1α) or on a Ventana automated slide stainer in combination with the iView DAB detection kit and accessories according to manufacturer’s instructions. Primary antibodies were omitted in negative controls. Nuclei were counterstained with Mayer’s haematoxylin (Sigma-Aldrich). Image screening and photography were performed using a Leica microscope IM1000 System.

### Animal studies approval

The experiments with animals were performed in compliance with the institutional guidelines and approved by the Local Committee for ‘Good animal experimental activities’. The experimental protocol was approved by the Animal Care and Use Committee of Università della Campania “L. Vanvitelli” (1966/7.17.2012). Animal care complied with Italian regulations on protection of animals used for experimental and other scientific purposes (116/1992) as well as with the EU guidelines for the use of experimental animals (2010/63/EU). Mice were housed in the Animal Facility of Università della Campania “L. Vanvitelli”.

### Statistical analysis

To analyse differences in HIF3α expression/methylation, statistical significance was calculated through a paired two-tailed t-test using the SPSS 17 software (SPSS, Inc. Chicago, IL, USA). Data are expressed as mean ± SEM of three biological replicates. *p*-values < 0.05 were considered significant.

### Data availability

All data generated or analysed during this study are included in this published article (and Suppl. Information files). For layout reasons, some data generated were modified, but raw data are included in Suppl. Information files. The datasets generated during and/or analysed during the current study are available from the corresponding author on reasonable request.

## Electronic supplementary material

Supplementary Information
