# Author Correction: Pro-inflammatory cytokines activate hypoxia-inducible factor 3α via epigenetic changes in mesenchymal stromal/stem cells

**DOI:** 10.1038/s41598-020-62861-8

**Published:** 2020-04-17

**Authors:** Francesca Cuomo, Antonietta Coppola, Chiara Botti, Ciro Maione, Amalia Forte, Lucia Scisciola, Giuseppina Liguori, Ilaria Caiafa, Matilde Valeria Ursini, Umberto Galderisi, Marilena Cipollaro, Lucia Altucci, Gilda Cobellis

**Affiliations:** 1 Department of Biochemistry, Biophysics and General Pathology, Università degli Studi della Campania L. Vanvitelli, Via L. De Crecchio, 7, 80138 Naples, Italy; 2 Department of Experimental Medicine, Università degli Studi della Campania L. Vanvitelli, Via L. De Crecchio, 7, 80138 Naples, Italy; 3 Istituto Nazionale Tumori, Struttura Complessa Oncologia Medica Melanoma Immunoterapia Oncologica e Terapia Innovativa, Via M. Semmola, 80131 Naples, Italy; 4 Institute of Genetics and Biophysics, ‘A. Buzzati-Traverso’ (IGB), via P. Castellino, 111, 80131 Naples, Italy; 5 Present Address: Laboratorio di Patologia Clinica, Ospedale Santobono, Via M. Fiore 6, 80129 Naples, Italy

Correction to: *Scientific Reports* 10.1038/s41598-018-24221-5, published online 11 April 2018

The original version of this Article contained errors.

In Figure 1b, panels for hypoxia, image for “scr siRNA” was a duplicate of image for “---”.

Additionally, in Figure 3b, as a result of figure misassembly all images for controls for amplicon #1 and amplicon #2 were duplicates. This panel has been corrected. The original data for these images was provided in the Supplementary File, which has been updated to show which sections of the original data were used to assemble main figures.

These errors have been corrected in the PDF and HTML versions of the Article, and in the accompanying Supplementary Information file.

