# Supplementary material for: Pro-inflammatory cytokines activate hypoxia-inducible factor 3α via epigenetic changes in mesenchymal stromal/stem cells
Source: Sci Rep. 2018 Apr 11;8:5842. doi: 10.1038/s41598-018-24221-5 (PMC5895792; doi:10.1038/s41598-018-24221-5)

(# SREP-17-07283B)

Pro-inflammatory cytokines activate hypoxia-inducible factor 3a via epigenetic changes in mesenchymal stromal/stem cells

Francesca Cuomo[a], Antonietta Coppola[a], Chiara Botti[a,§], Ciro Maione[a], Amalia Forte[b], Lucia Scisciola[a], Giuseppina Liguori[c], Ilaria Caiafa[a], Matilde Valeria Ursini[d], Umberto Galderisi[b], Marilena Cipollaro[b], Lucia Altucci[a] and Gilda Cobellis[a]

a

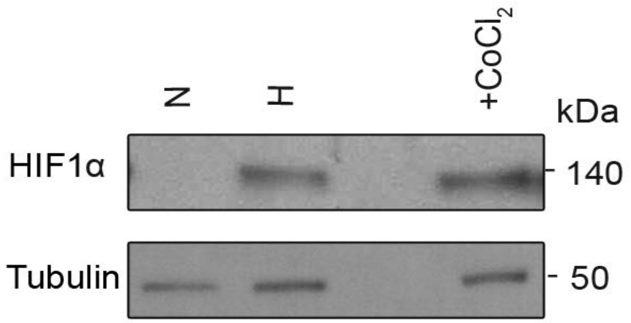

b

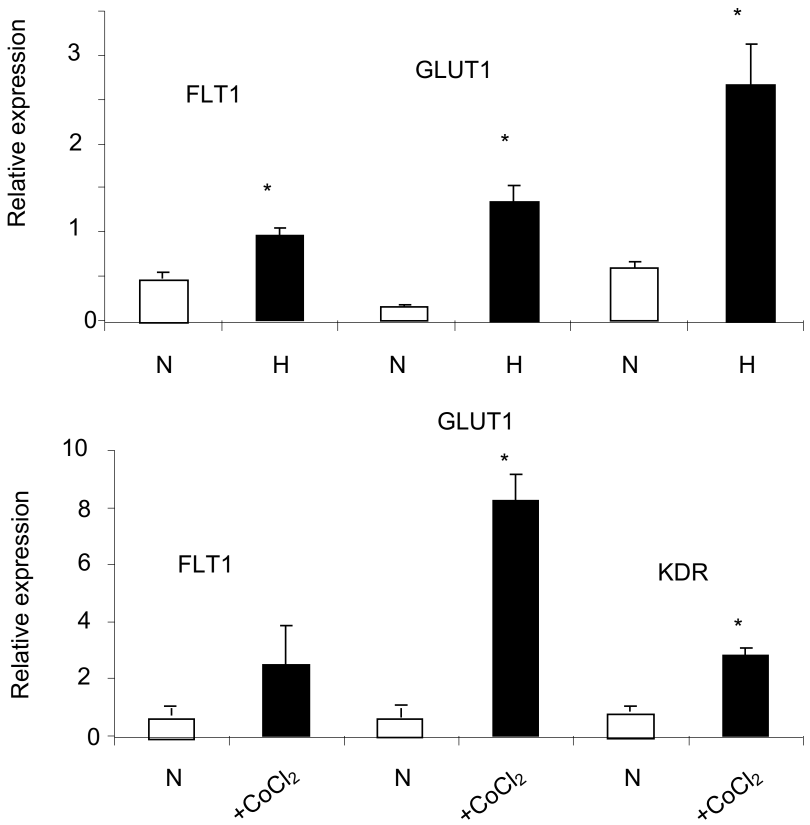

Cuomo et al Suppl. Figure 1

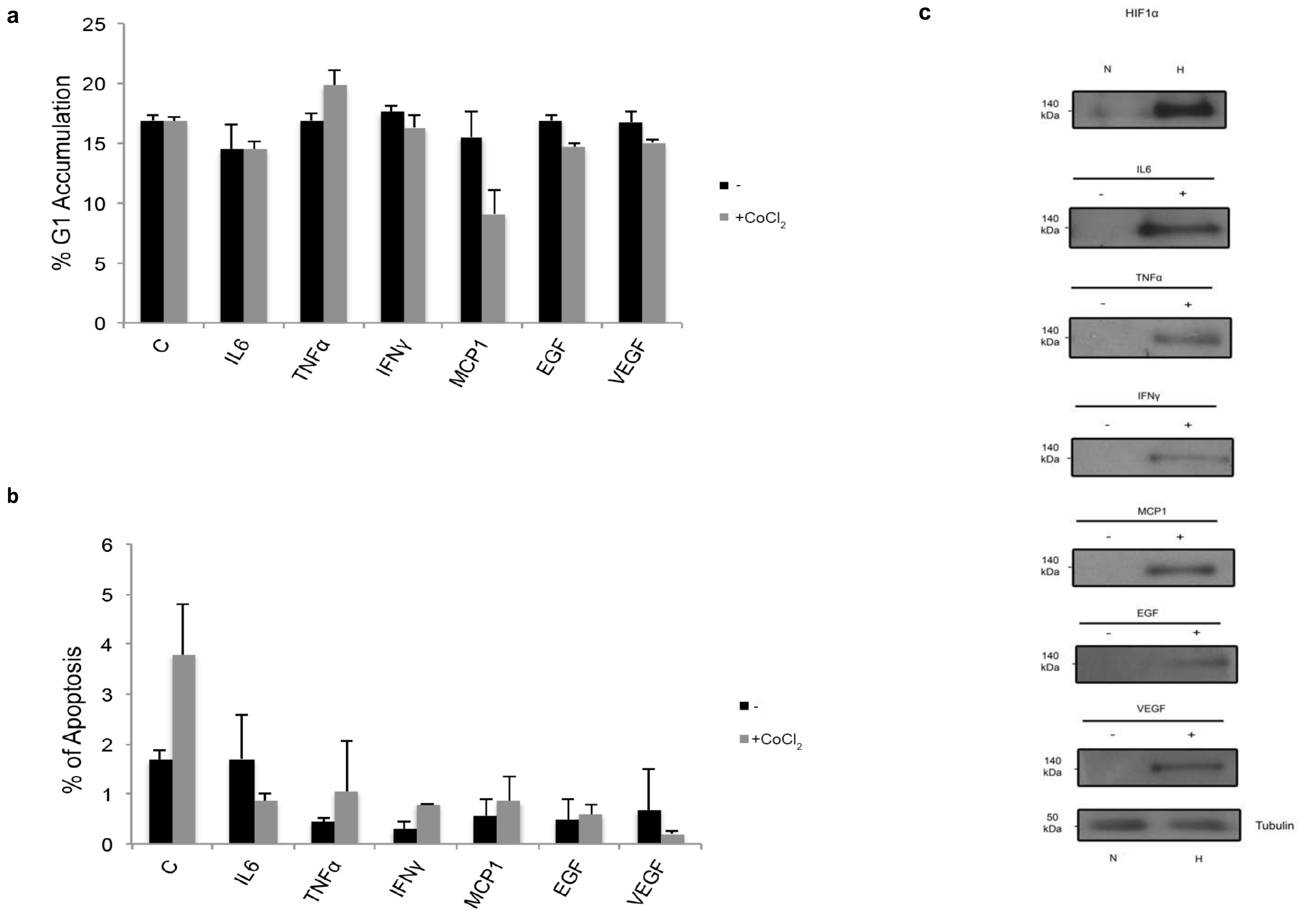

Cuomo et al Suppl. Figure 2

a

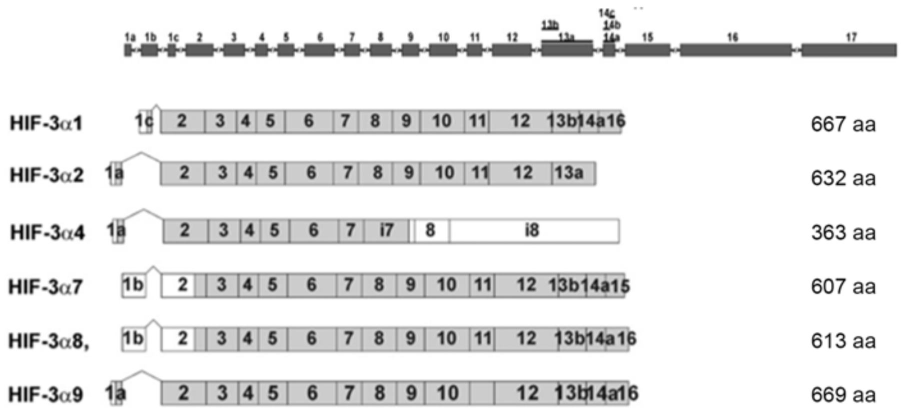

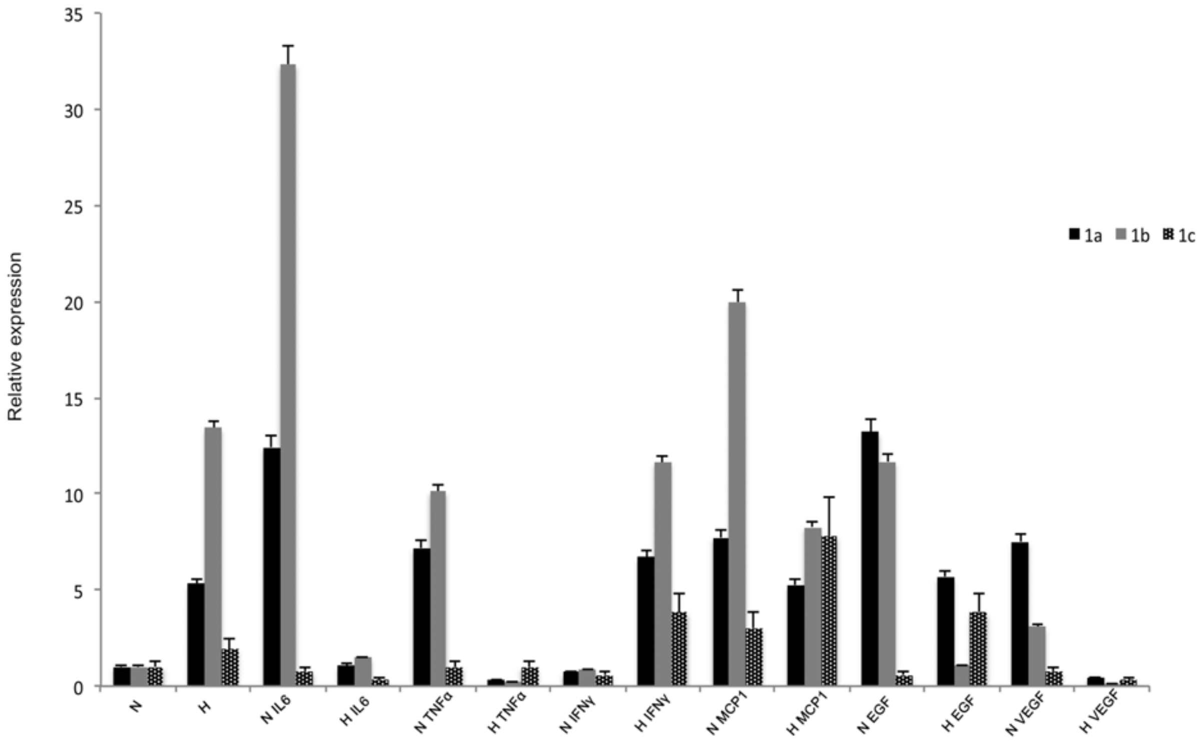

Cuomo et al Suppl. Figure 3

a

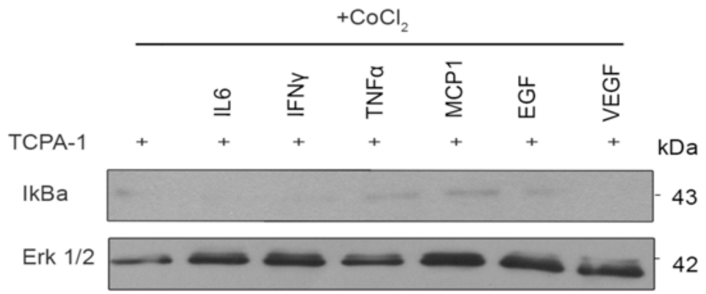

b

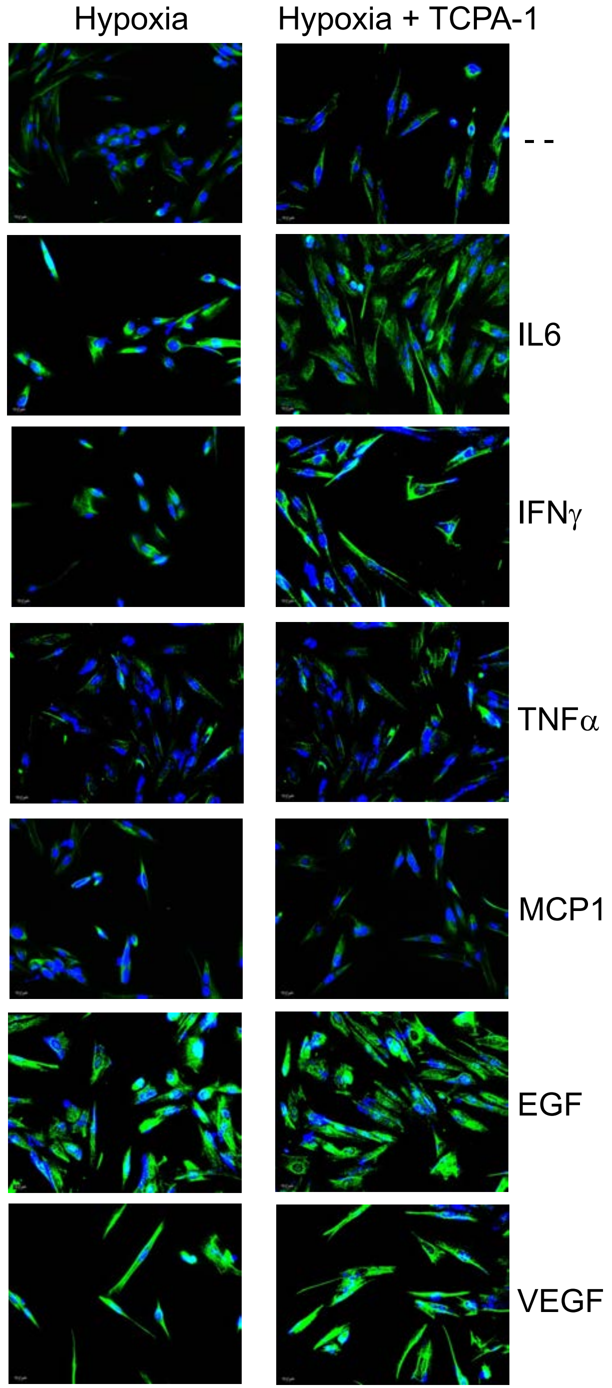

Cuomo et al Suppl. Figure 4

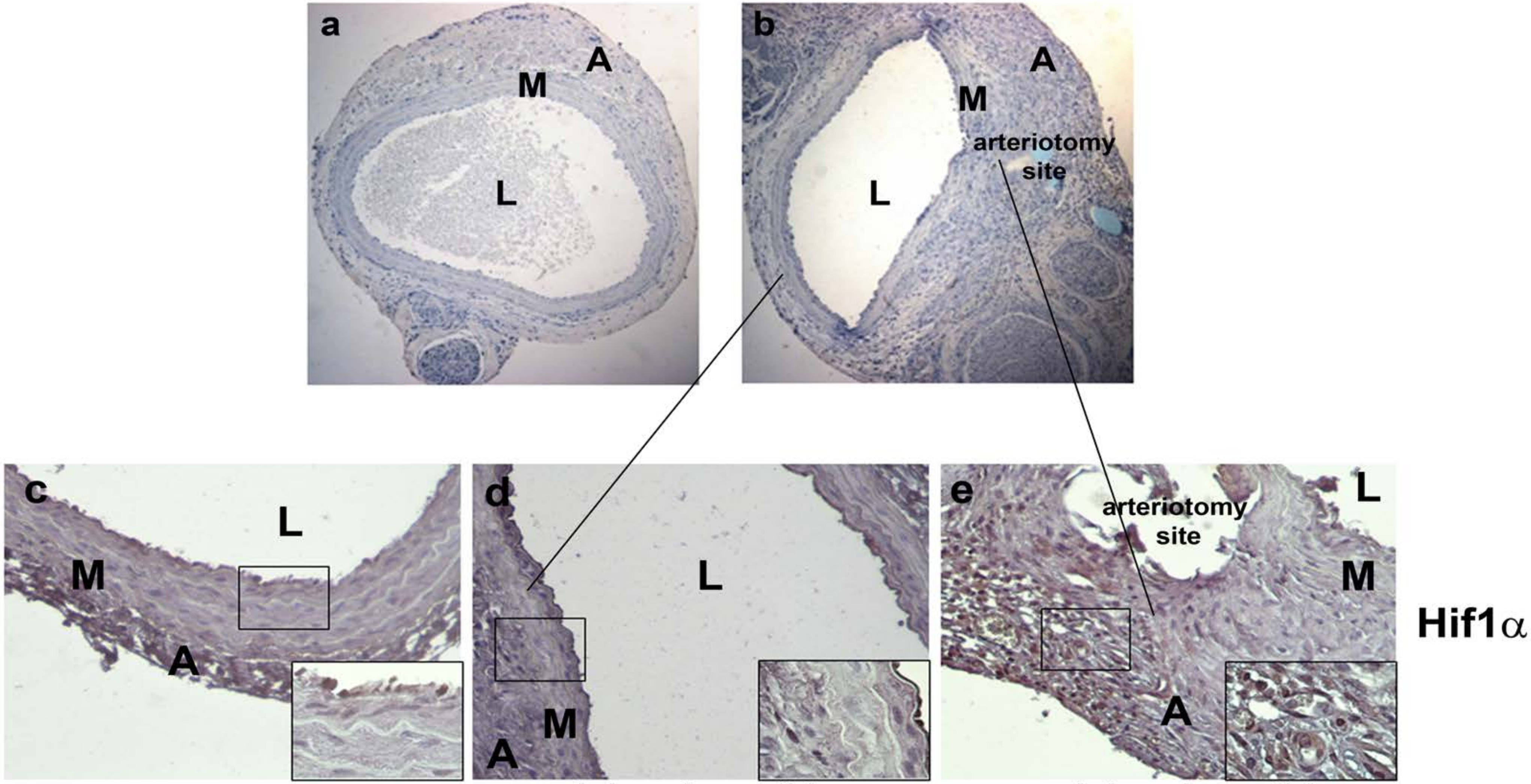

Uninjured carotid

Injured carotid
Region distal to
arteriotomy site

Injured carotid
Region proximal to
arteriotomy site

Cuomo et al Suppl. Figure 5

Supplementary Figure Legends

Supplementary Figure 1: a) Immunoblotting analysis of HIF1a protein in hMSC cells cultured either in 1%O2 conditions generated by GasPack method (H) or in presence of CoCl2 for 24 h. Total proteins were extracted and WB probed with antibodies against HIF1α. Tubulin was used as loading control. Protein molecular weights are shown. b) Expression analysis of FLT1, GLUT1 and KDR mRNAs in hMSC cells cultured either in 1%O2 conditions generated by GasPack method (H) or in presence of CoCl2 for 24 h. Gene expression data are reported as 2-DDCt method, normalized to housekeeping gene (beta-actin mRNA) and ALU sequences. Data are expressed as means ± SEM. Statistical significance is reported: *: $p < 0.05$

Supplementary Figure 2: a) G1-phase accumulation: hMSCs were cultured in normoxic (N) and hypoxic (H) conditions and treated with IL6, TNFα, IFNγ MCP1, EGF, and VEGF for 24 h and analysed by flow cytometry using CellQuest software. b) Quantification of apoptotic cells in pre-G1 phase was performed by ModFit software. c) Immunoblotting analysis of HIF1a protein in hMSC cells cultured either in normoxic conditions or in presence of CoCl2 for 24 h in absence or in presence of indicated cytokines. Total proteins were extracted and WB probed with antibodies against HIF1α. Tubulin was used as loading control. Protein molecular weights are shown. Images derived from different part of the same gel and cropped for layout reasons and included in Supplementary Information.

Supplementary Figure 3: a) Schematic representation of the HIF3A locus and relative isoforms, modified from[15].

Supplementary Figure 4: a) Immunoblotting analysis of IkBa in hMSCs cultured in CoCl2- induced hypoxia conditions and treated with IL6, IFNg, TNFα, MCP1, EGF and VEGF for 24h, after an incubation with TCPA-1 for 1hr. b) Immunofluorescence analysis of HIF3a protein in hMSCs cultured in hypoxia in absence and in presence of indicated cytokines and probed with antibodies against HIF3a. Cells were pre-treated with TCPA-1 for 1h before cytokines supplementation. Scale bars: 10μm)

Supplementary Figure 5: Immunohistochemical analysis of Hif1α expression in uninjured rat carotids and in carotids from Wistar male rats harvested 7 days after arteriotomy. a: uninjured rat carotid; b: arteriotomy-injured rat carotid harvested 7 days after injury, haematoxylin staining. The injury site is indicated in b, where arteriotomy is followed by the application of an 8.0 polypropylene stitch (light blue); c-e: representative immunohistochemical staining of Hif1α in uninjured rat carotid (c) and in injured carotids harvested 7 days after arteriotomy, showing a carotid region distal (d) and proximal (e) to the arteriotomy site. a,b: 10x magnification; c-e: 20x magnification; small insets: 40x magnification of selected areas enclosed in black rectangles, representative of nuclei positive to Hif1α in intima, adventitia, *vasa vasorum* and perivascular tissue. Brown staining corresponds to target protein expression. Nuclei were counterstained with haematoxylin. L: lumen; M: media; A: adventitia.

Supplementary Figure 7: Raw data (8 panels) from MS-PCR experiments used for figure 3B

Supplementary Figure 8: Raw data (8 panels) from replicate MS-PCR experiment.

Raw data of HIF1a expression in normoxic, hypoxic and $CoCl_2$-induced hypoxia in Suppl. Figure 2

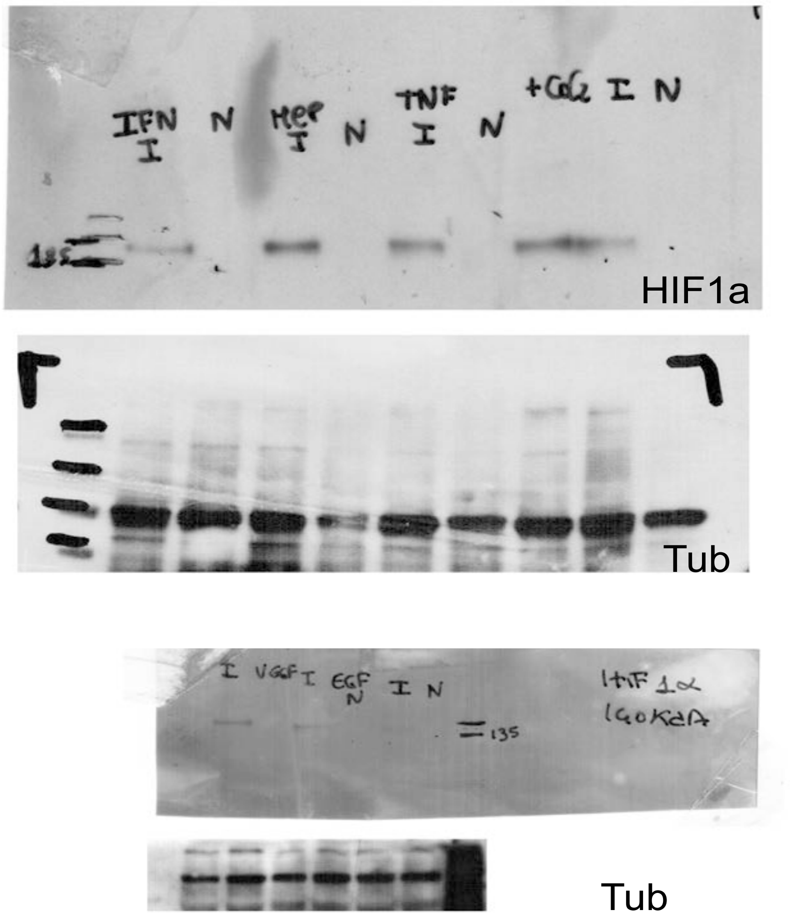

Raw data from MS-PCR experiments used for figure 3B

INPUT 1

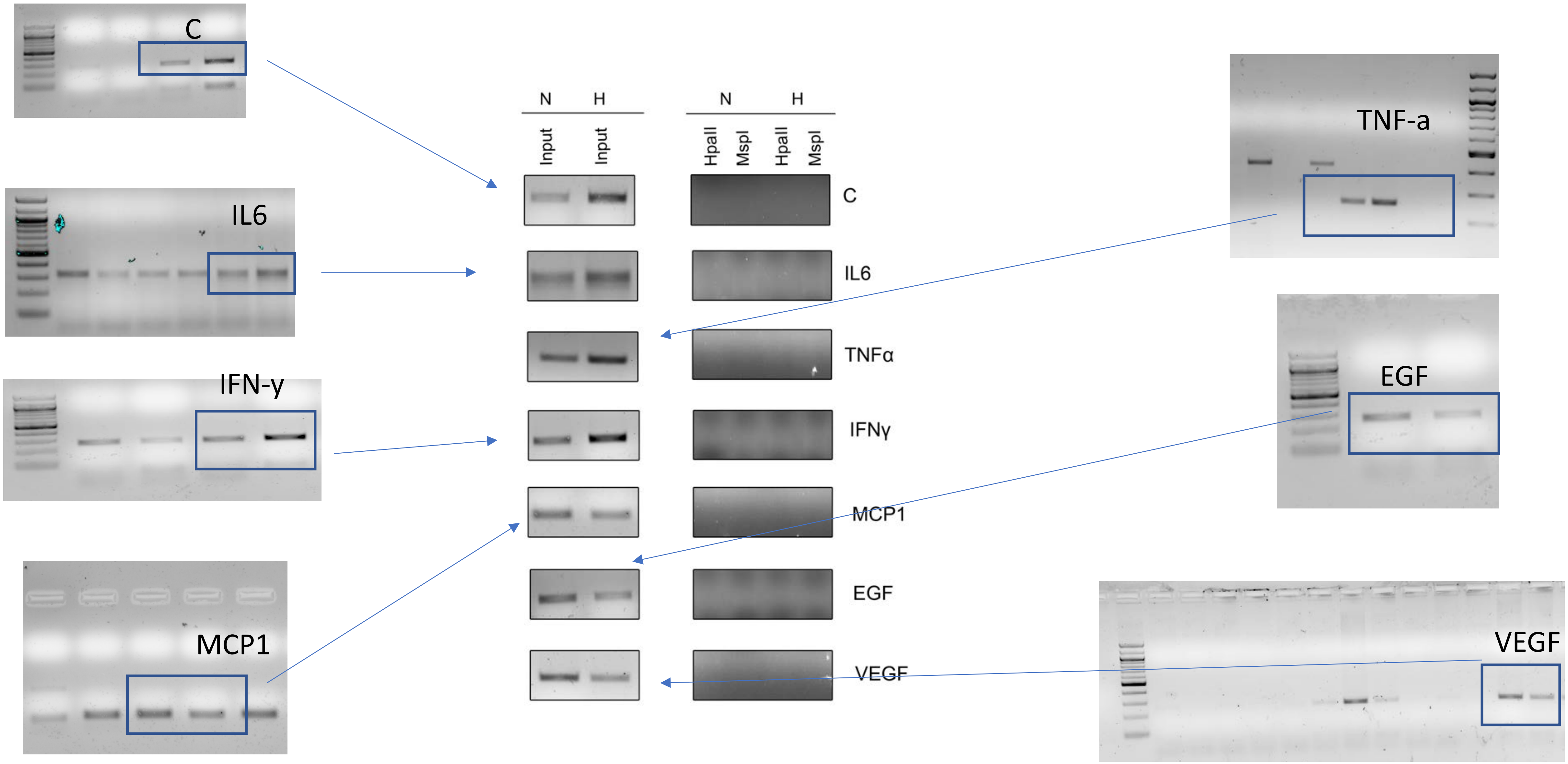
C
IL6
IFN-y
MCP1
N
H
Input
Input
N
H
HpaII
MspI
HpaII
MspI
C
IL6
TNFα
IFNγ
MCP1
EGF
VEGF
TNF-a
EGF
VEGF

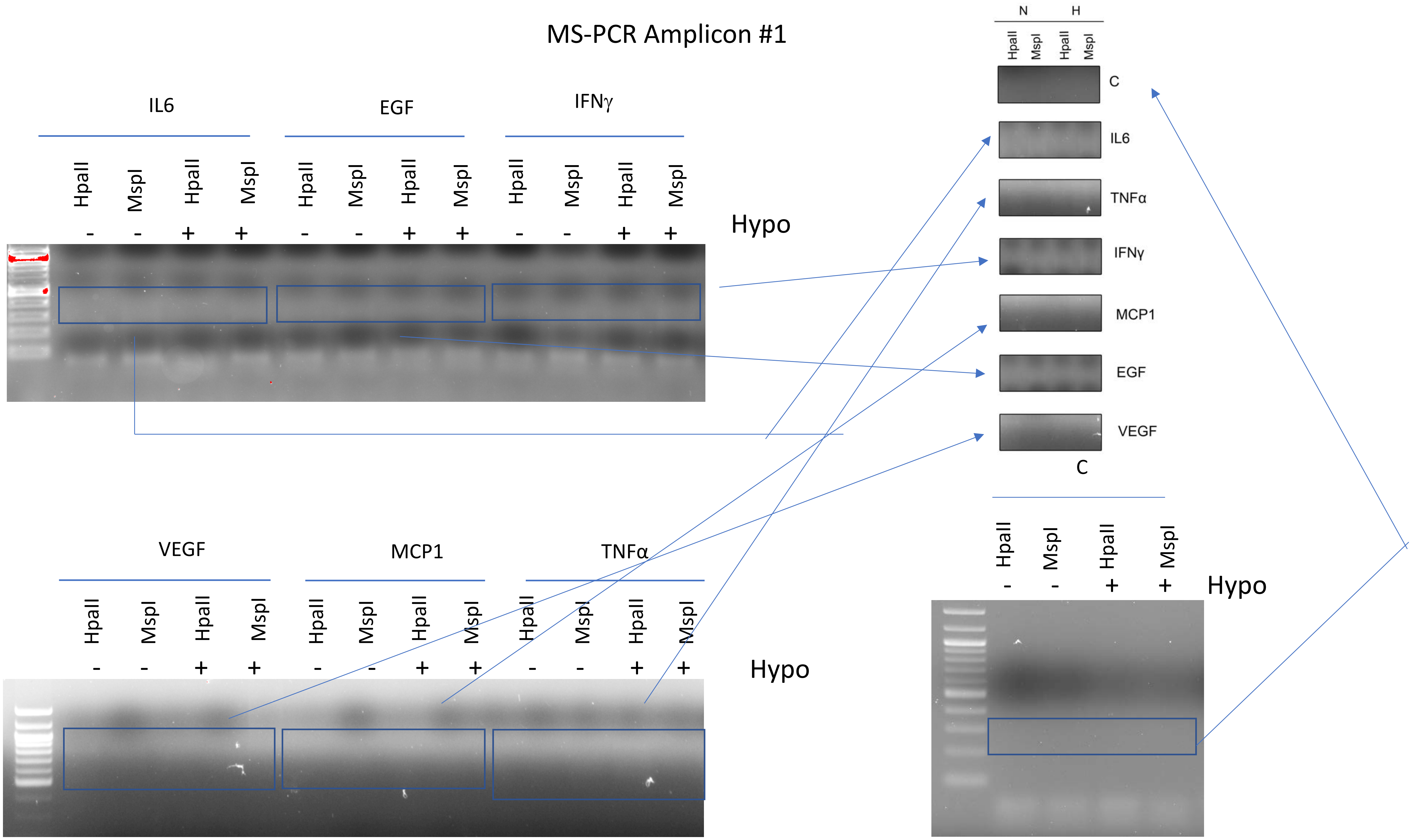
MS-PCR Amplicon #1
IL6
EGF
IFNγ
HpaII MspI HpaII MspI HpaII MspI HpaII MspI HpaII MspI HpaII MspI
- - + + - - + + - - + +
Hypo
VEGF
MCP1
TNFα
HpaII MspI HpaII MspI HpaII MspI HpaII MspI HpaII MspI HpaII MspI
- - + + - - + + - - + +
Hypo
N
H
HpaII MspI HpaII MspI
C
IL6
TNFα
IFNγ
MCP1
EGF
VEGF
C
HpaII MspI HpaII MspI
- - + +
Hypo

INPUT 2

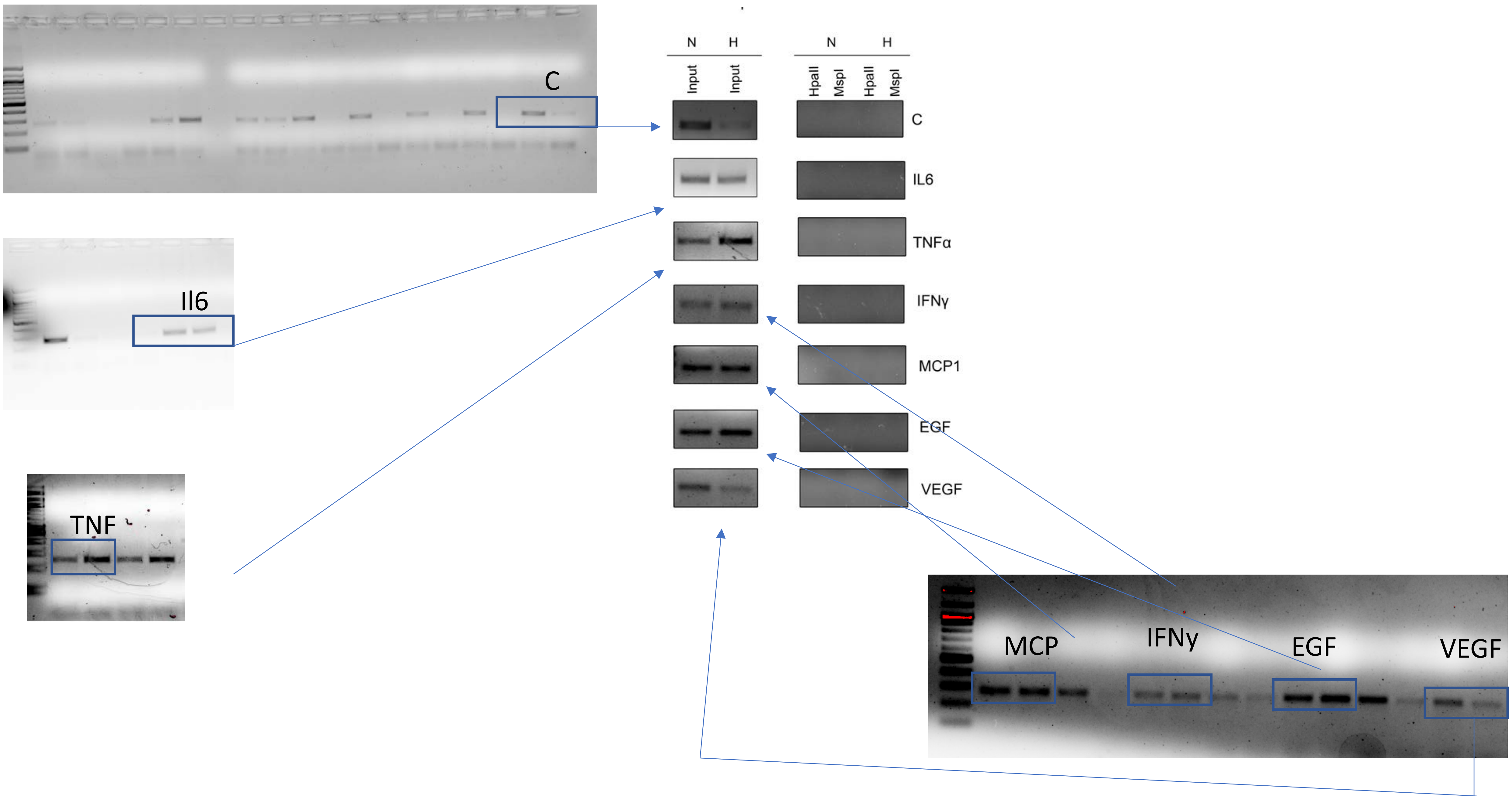
C
Il6
TNF
N
H
Input
Input
N
H
HpaII
MspI
HpaII
MspI
C
IL6
TNFα
IFNγ
MCP1
EGF
VEGF
MCP
IFNγ
EGF
VEGF

MS-PCR Amplicon #2

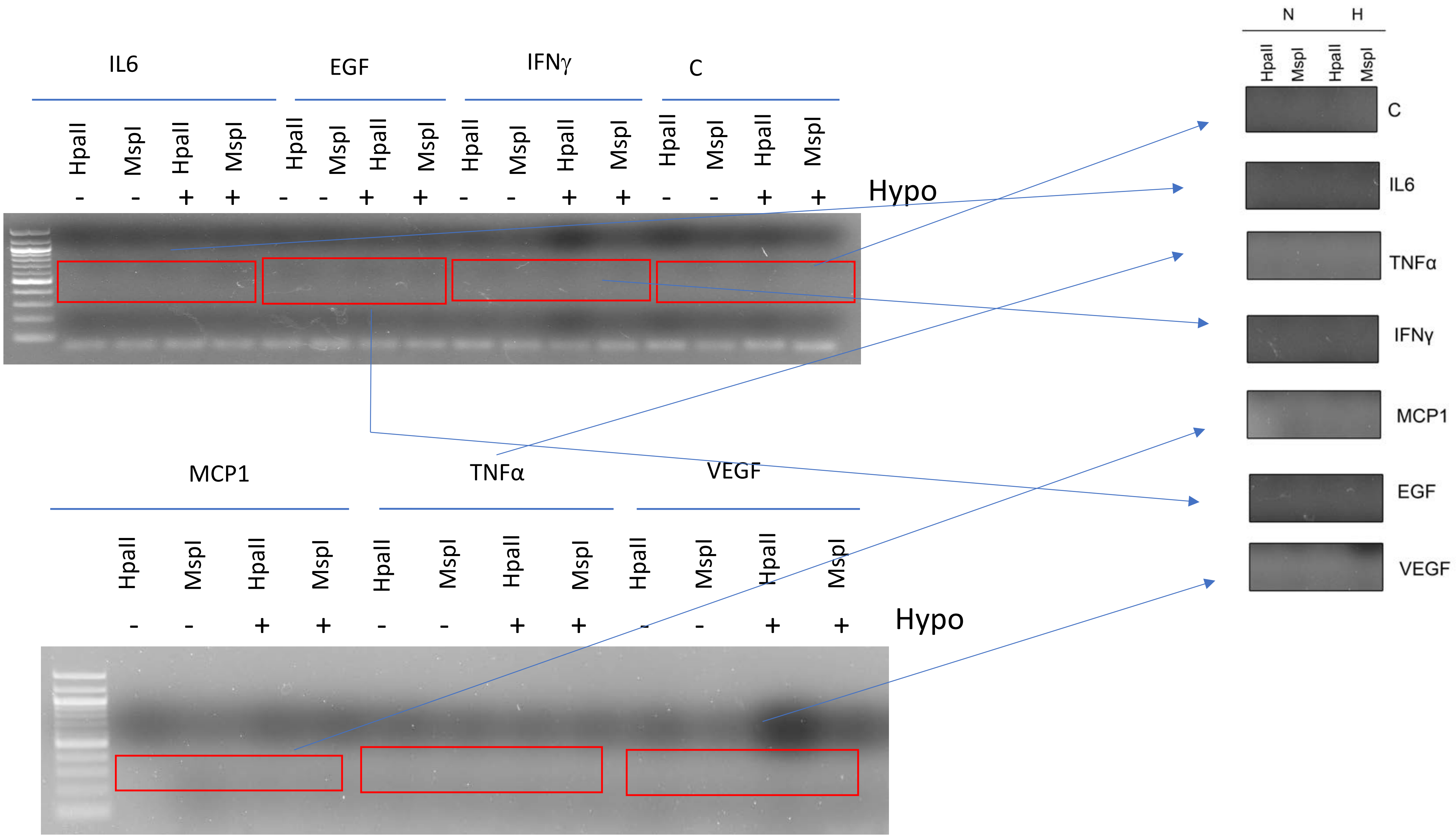

INPUT 3

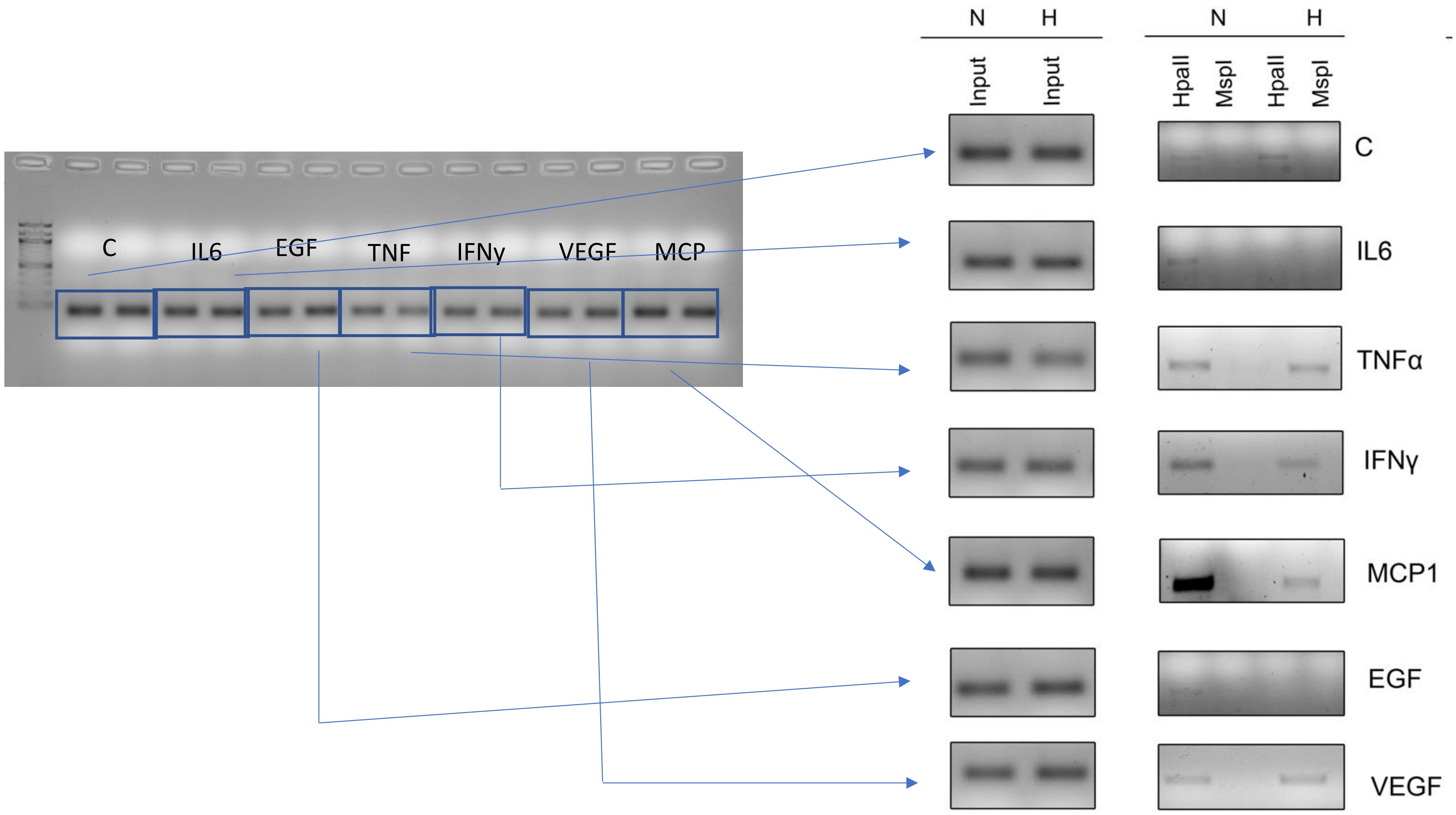

C
IL6
EGF
TNF
IFNγ
VEGF
MCP
N
H
Input
Input
N
H
HpaII
MspI
HpaII
MspI
C
IL6
TNFα
IFNγ
MCP1
EGF
VEGF

MS-PCR Amplicon #3

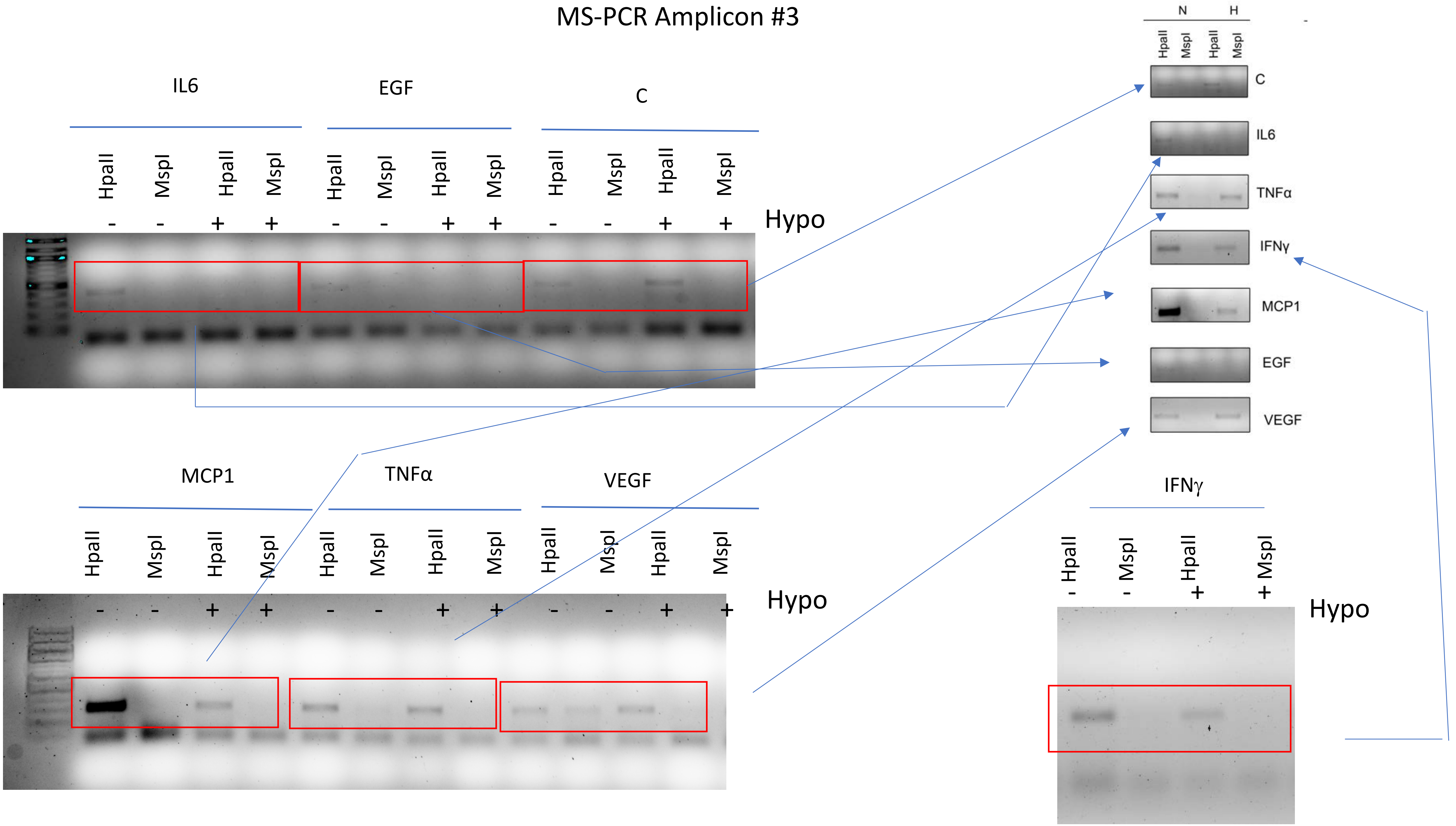

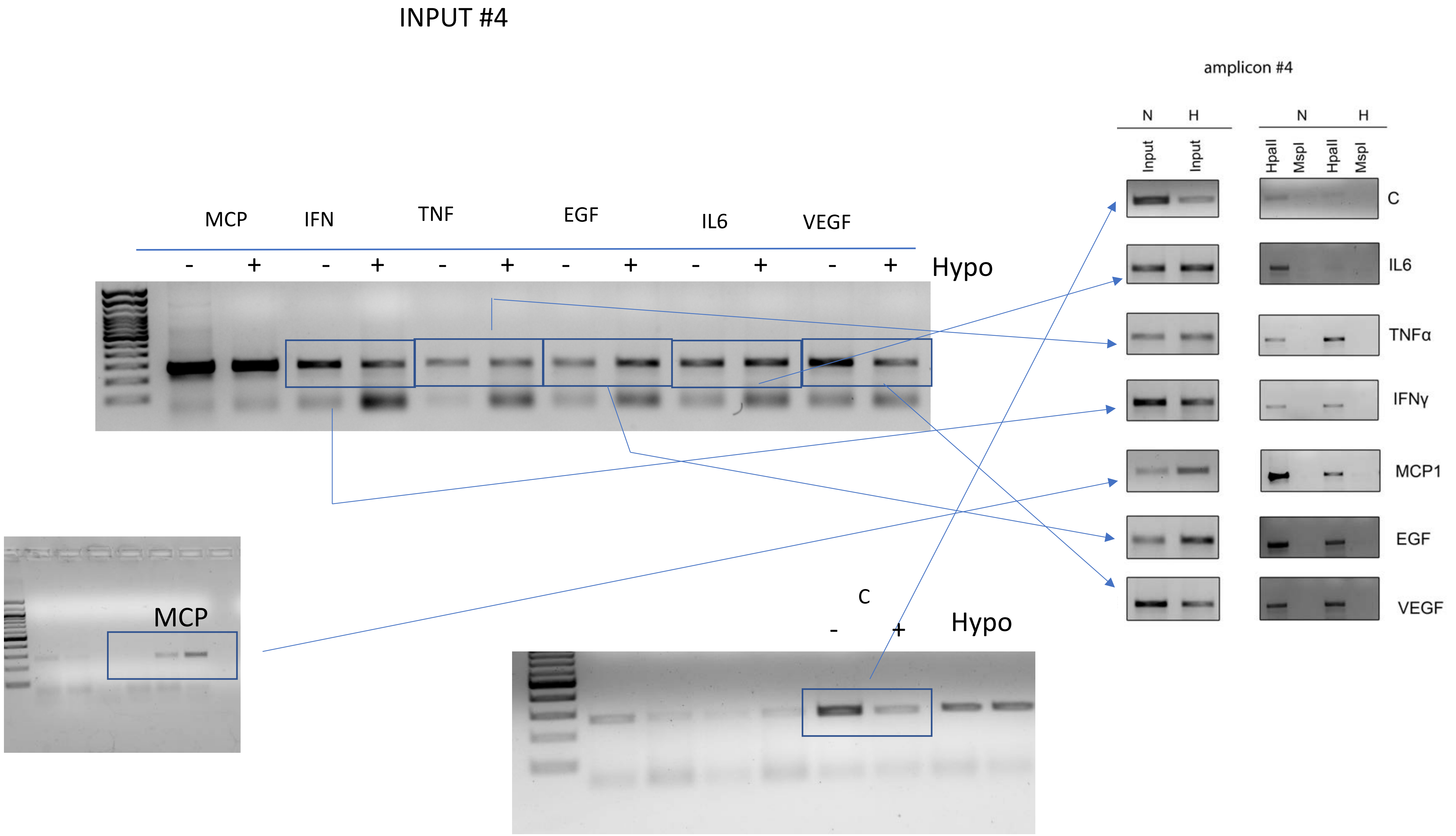
INPUT #4
MCP
IFN
TNF
EGF
IL6
VEGF
- + - + - + - + - + - +
Hypo
amplicon #4
N H
Input Input
N H
HpaII MspI HpaII MspI
C
IL6
TNFα
IFNγ
MCP1
EGF
VEGF
MCP
C
- +
Hypo

MS-PCR Amplicon #4

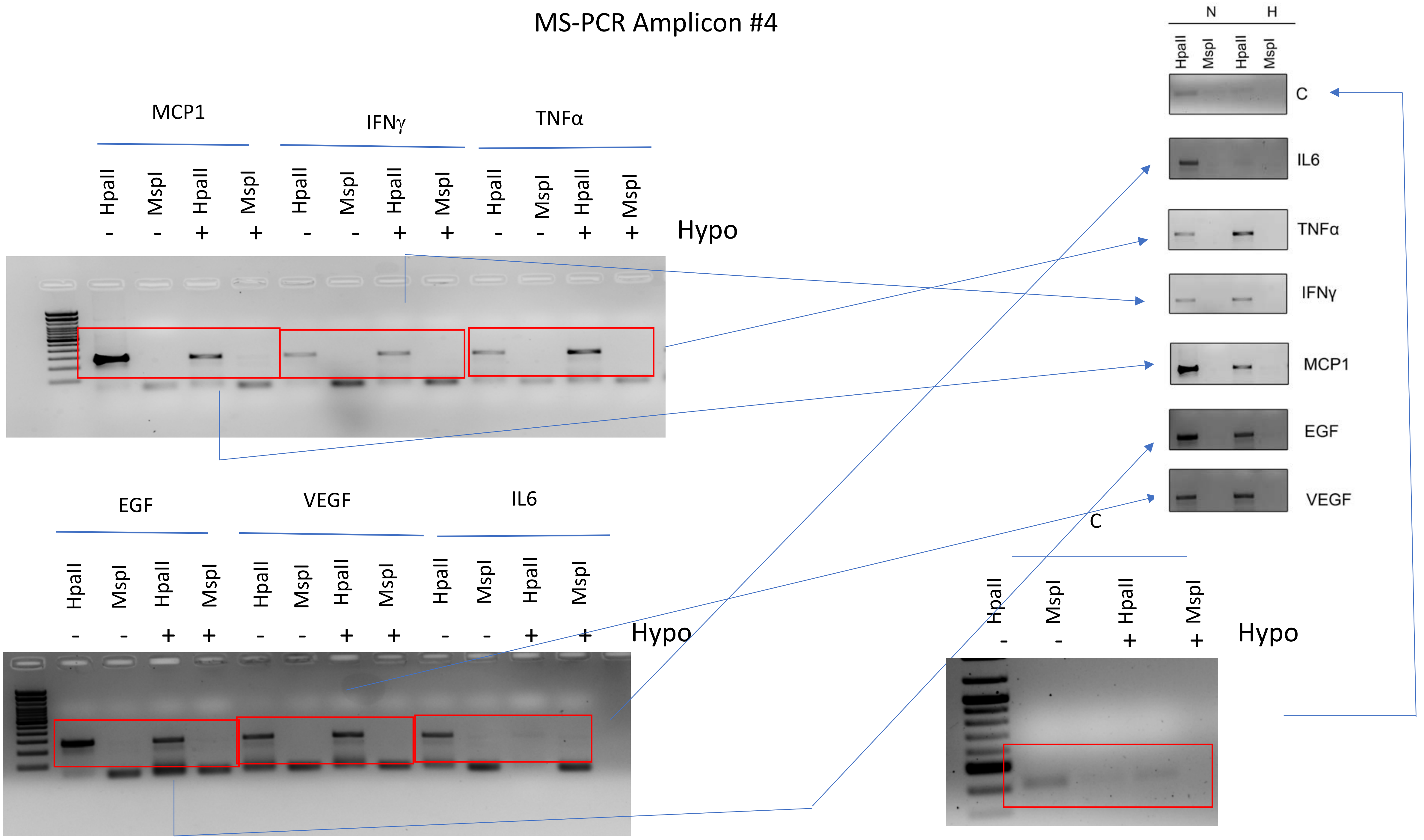

Raw Replicate data from MS-PCR experiment

AMPLICON INPUT #1

IL6 IFNγ TNFα MCP1 EGF VEGF

\- + - + - + - + - + - +

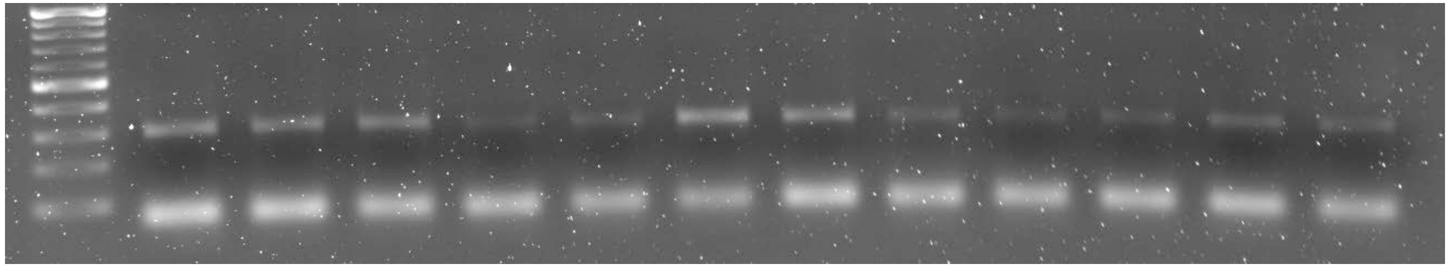

AMPLICON INPUT #2

C IL6 IFNγ TNFα MCP1 EGF VEGF

\- + - + - + - + - + - + - +

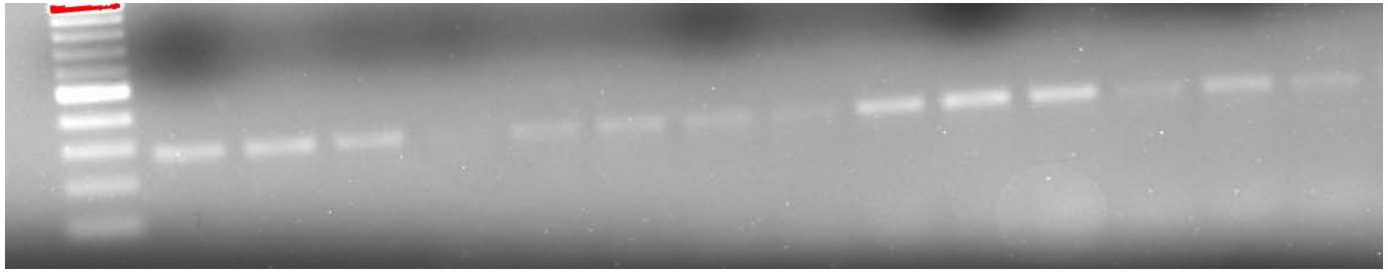

Raw Replicate data from MS-PCR experiment

INPUT #3

C IL6 IFNγ TNFα MCP1 EGF VEGF

- + - + - + - + - + - + - +

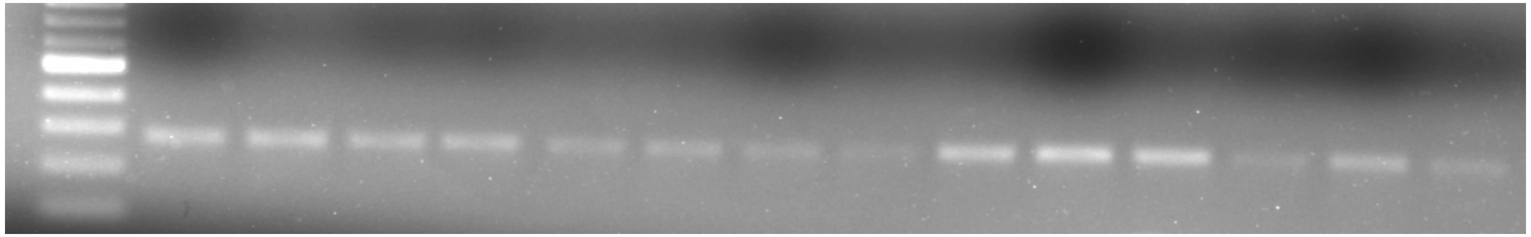

INPUT #4

C IL6 IFNγ TNFα MCP1 EGF VEGF

- + - + - + - + - + - + - +

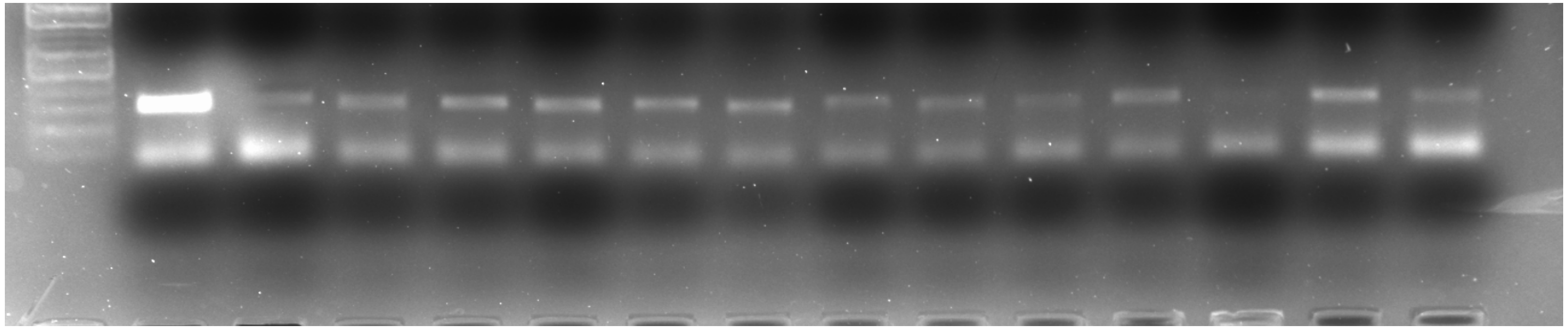

Raw Replicate data from MS-PCR experiment

INPUT CNTR #1#2 #3 #4

#2 #1 #4 #3

- + - + - + - +

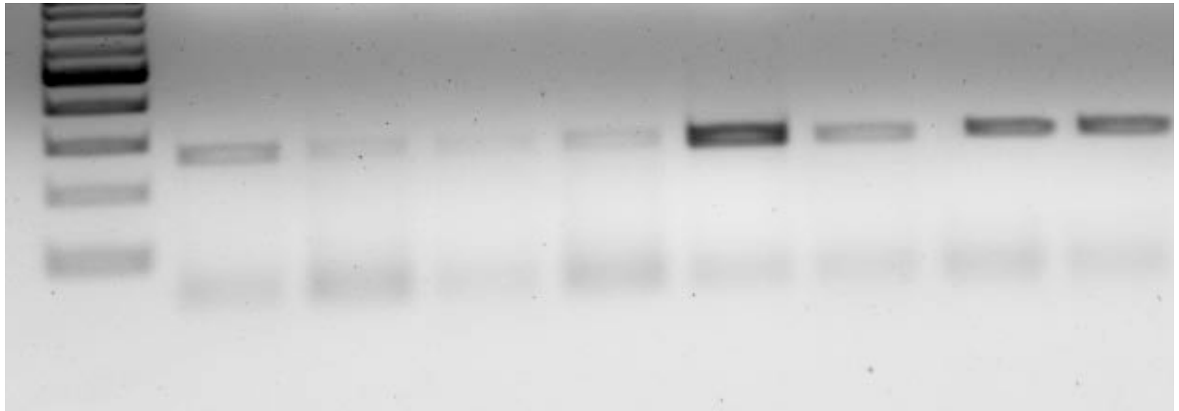

Raw Replicate data from MS-PCR experiment

#1 #2

HpaII MSP1 HpaII MSP1 HpaII MSP1 HpaII MSP1

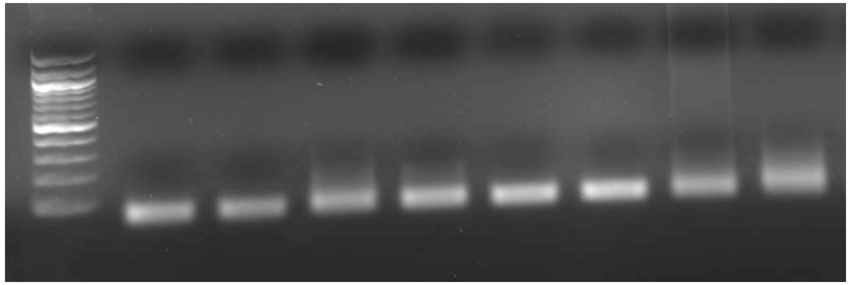

#3

HpaII MSP1 HpaII MSP1

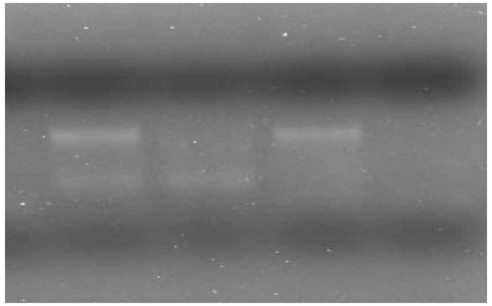

#4

HpaII MSP1 HpaII MSP1

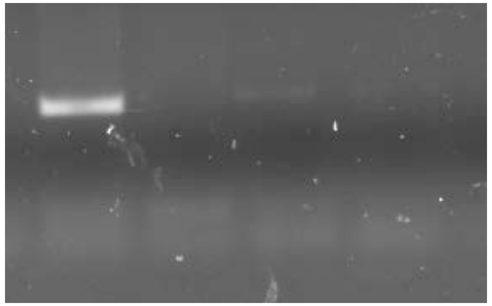

Raw Replicate data from MS-PCR experiment: amplicon #1

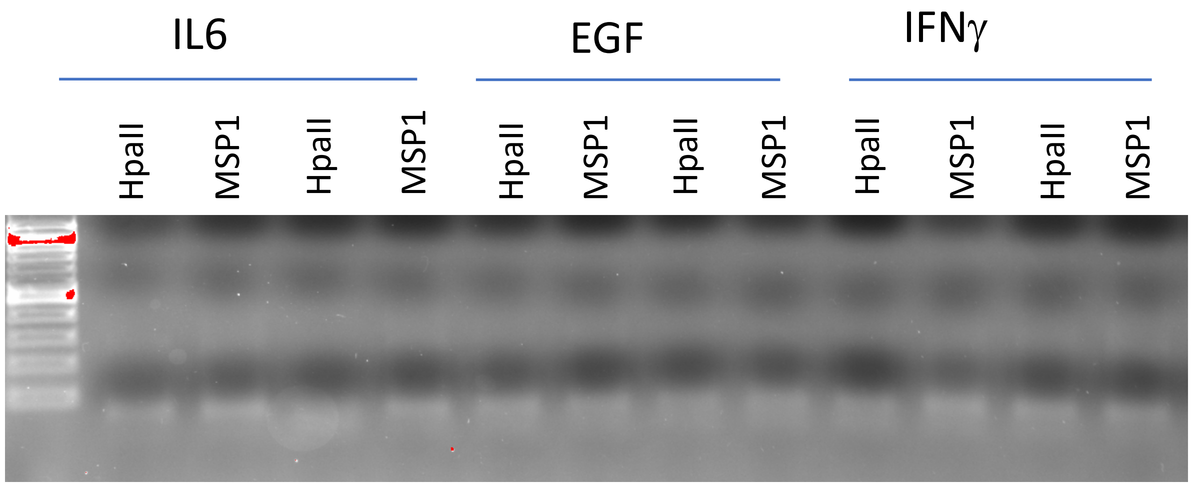

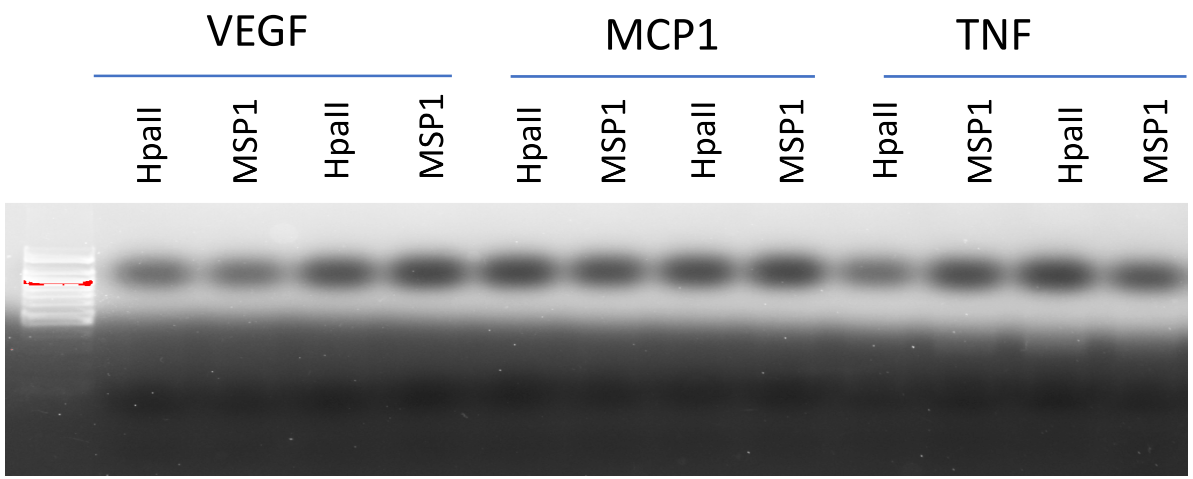

Raw Replicate data from MS-PCR experiment: amplicon #2

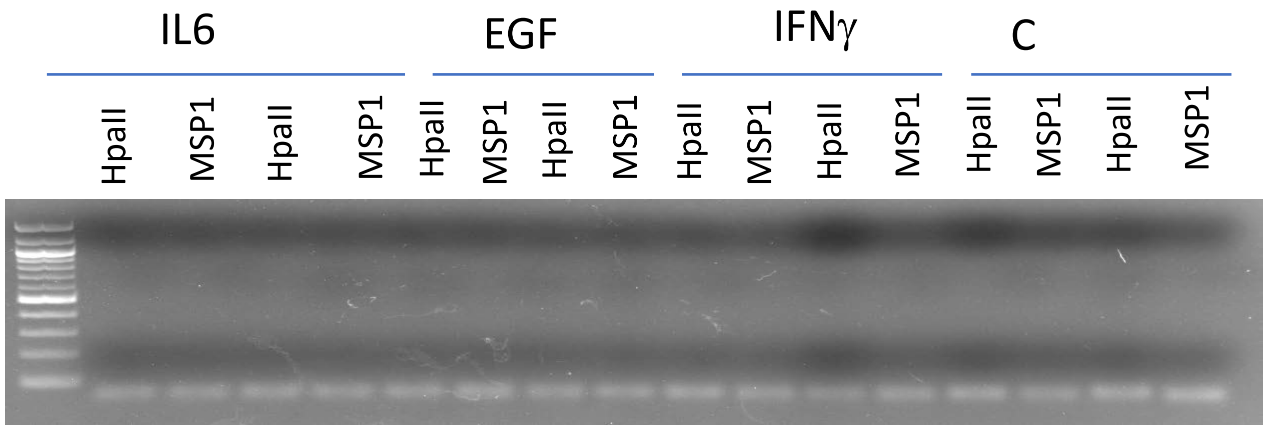

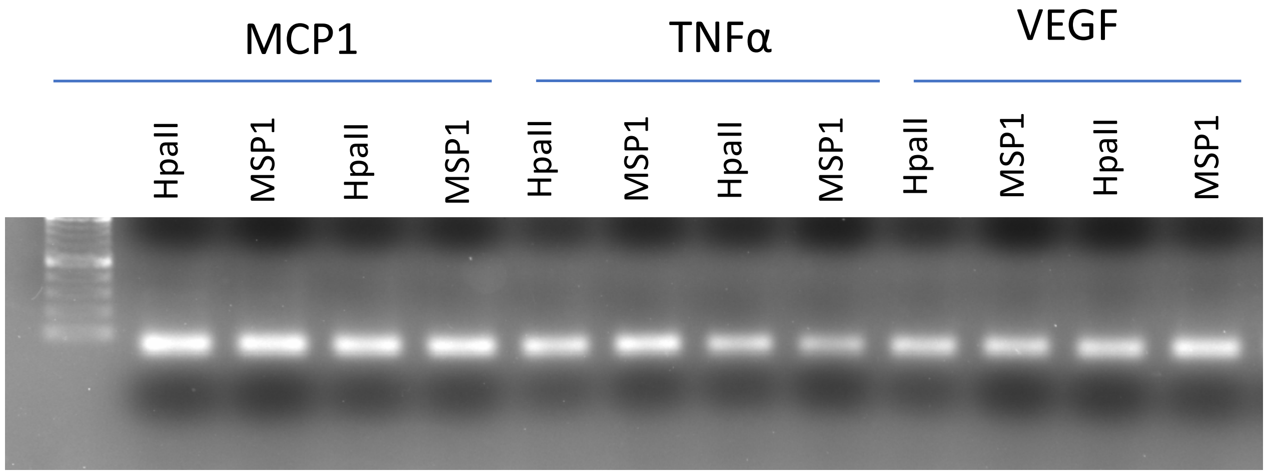

Raw Replicate data from MS-PCR experiment: amplicon #3

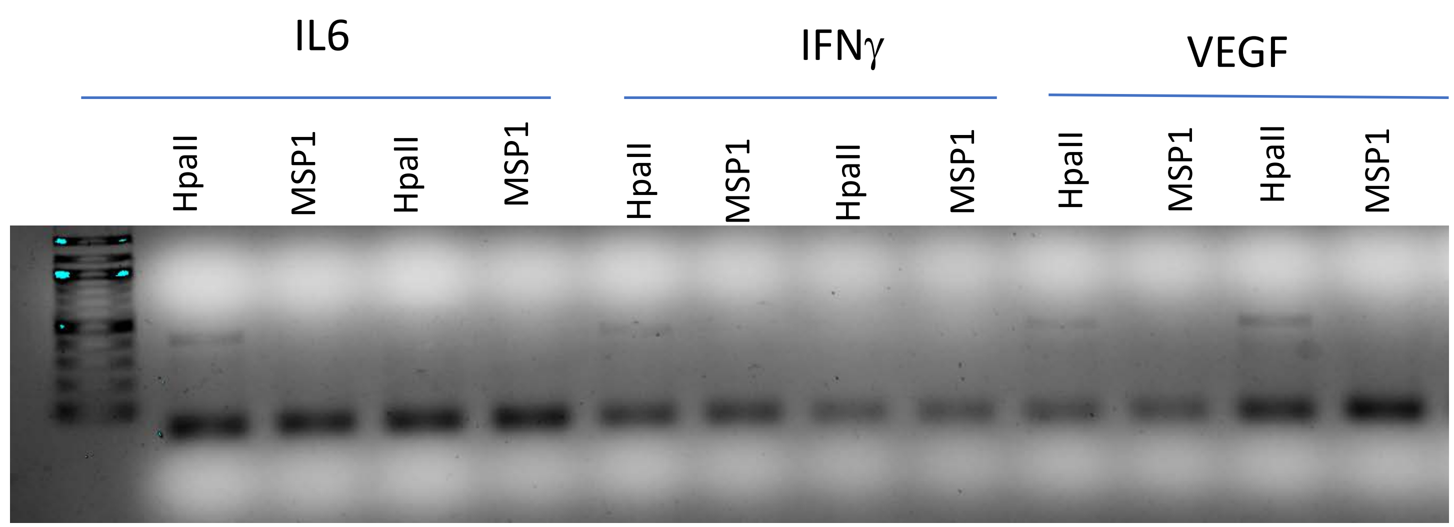

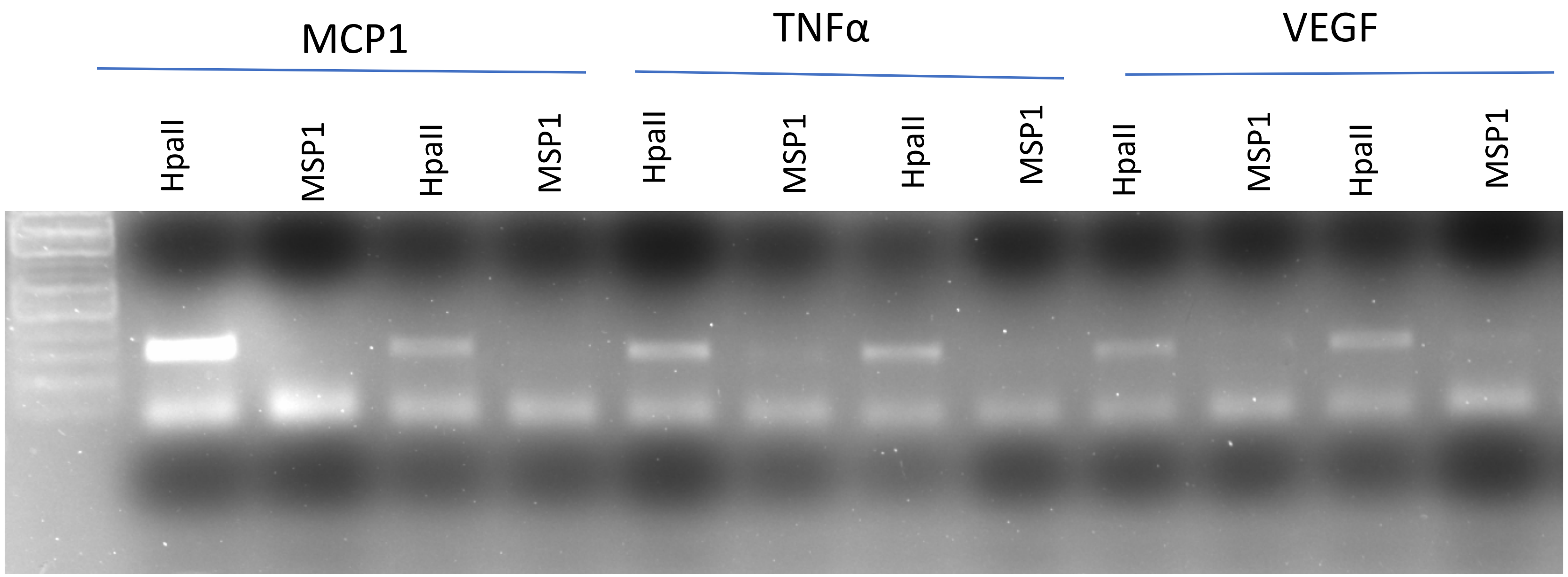

Raw Replicate data from MS-PCR experiment: amplicon #4

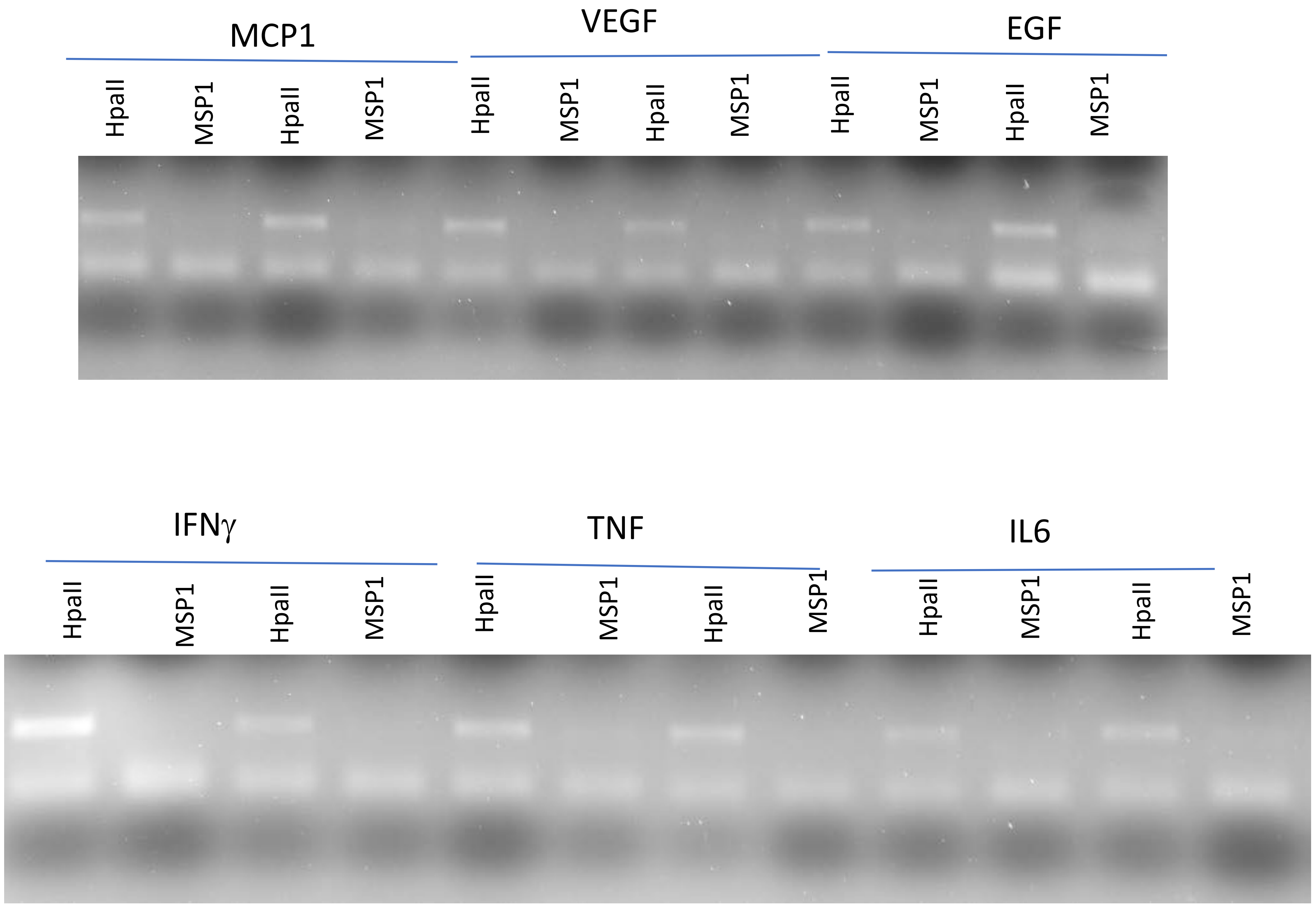

Supplement: Supplementary file 1 — Supplementary Information [file 41598_2018_24221_MOESM1_ESM.pdf]
